# Coincident glutamatergic depolarizations enhance GABA_A_ receptor-dependent Cl^-^ influx in mature and suppress Cl^-^ efflux in immature neurons

**DOI:** 10.1371/journal.pcbi.1008573

**Published:** 2021-01-19

**Authors:** Aniello Lombardi, Peter Jedlicka, Heiko J. Luhmann, Werner Kilb

**Affiliations:** 1 Institute of Physiology, University Medical Center Mainz, Johannes Gutenberg University, Mainz, Germany; 2 ICAR3R - Interdisciplinary Centre for 3Rs in Animal Research, Faculty of Medicine, Justus-Liebig-University, Giessen, Germany; 3 Institute of Clinical Neuroanatomy, Neuroscience Center, Goethe University, Frankfurt/Main, Germany; 4 Frankfurt Institute for Advanced Studies, Frankfurt am Main, Germany; Inria, FRANCE

## Abstract

The impact of GABAergic transmission on neuronal excitability depends on the Cl^-^-gradient across membranes. However, the Cl^-^-fluxes through GABA_A_ receptors alter the intracellular Cl^-^ concentration ([Cl^-^]_i_) and in turn attenuate GABAergic responses, a process termed ionic plasticity. Recently it has been shown that coincident glutamatergic inputs significantly affect ionic plasticity. Yet how the [Cl^-^]_i_ changes depend on the properties of glutamatergic inputs and their spatiotemporal relation to GABAergic stimuli is unknown. To investigate this issue, we used compartmental biophysical models of Cl^-^ dynamics simulating either a simple ball-and-stick topology or a reconstructed CA3 neuron. These computational experiments demonstrated that glutamatergic co-stimulation enhances GABA receptor-mediated Cl^-^ influx at low and attenuates or reverses the Cl^-^ efflux at high initial [Cl^-^]_i_. The size of glutamatergic influence on GABAergic Cl^-^-fluxes depends on the conductance, decay kinetics, and localization of glutamatergic inputs. Surprisingly, the glutamatergic shift in GABAergic Cl^-^-fluxes is invariant to latencies between GABAergic and glutamatergic inputs over a substantial interval. In agreement with experimental data, simulations in a reconstructed CA3 pyramidal neuron with physiological patterns of correlated activity revealed that coincident glutamatergic synaptic inputs contribute significantly to the activity-dependent [Cl^-^]_i_ changes. Whereas the influence of spatial correlation between distributed glutamatergic and GABAergic inputs was negligible, their temporal correlation played a significant role. In summary, our results demonstrate that glutamatergic co-stimulation had a substantial impact on ionic plasticity of GABAergic responses, enhancing the attenuation of GABAergic inhibition in the mature nervous systems, but suppressing GABAergic [Cl^-^]_i_ changes in the immature brain. Therefore, glutamatergic shift in GABAergic Cl^-^-fluxes should be considered as a relevant factor of short-term plasticity.

## Author summary

Information processing in the brain requires that excitation and inhibition are balanced. The main inhibitory neurotransmitter in the brain is gamma-amino-butyric acid (GABA). GABA actions depend on the Cl^-^-gradient, but activation of ionotropic GABA receptors causes Cl^-^-fluxes and thus reduces GABAergic inhibition. Here, we investigated how a coincident membrane depolarization by excitatory glutamatergic synapses influences GABA-induced Cl^-^-fluxes using a biophysical compartmental model of Cl^-^ dynamics, simulating either simple or realistic neuron topologies. We demonstrate that glutamatergic co-stimulation directly affects GABA-induced Cl^-^-fluxes, with the size of glutamatergic effects depending on the conductance, the decay kinetics, and localization of glutamatergic inputs. We also show that the glutamatergic shift in GABAergic Cl^-^-fluxes is surprisingly stable over a substantial range of latencies between glutamatergic and GABAergic inputs. We conclude from these results that glutamatergic co-stimulation alters GABAergic Cl^-^-fluxes and in turn affects the strength of GABAergic inhibition. These coincidence-dependent ionic changes should be considered as a relevant factor of short-term plasticity in the CNS.

## Introduction

Information transfer within networks of single neurons is carried out via synaptic contacts, that use different neurotransmitters acting on pre- and postsynaptic receptors. The two most important neurotransmitters in the mammalian brain are glutamate and γ-amino butyric acid (GABA), which in general exert an excitatory and inhibitory action in the postsynaptic cell, respectively [1]. Beside regulating the excitability of neuronal circuits, GABA is also required for the control of sensory integration, regulation of motor functions, generation of oscillatory activity, and neuronal plasticity [2]. The responses to GABA are mediated by metabotropic GABA_B_ receptors and by ionotropic GABA_A_ receptors, which are ligand-gated anion-channels with a high Cl^-^ permeability and a lower HCO_3_^-^ permeability [1]. The GABAergic effects therefore depend mainly on the Cl^-^ gradient across the neuronal plasma membrane [1, 3]. The typical inhibitory action of GABA requires a low intracellular Cl^-^ concentration ([Cl^-^]_i_), which is typically maintained by the action of a Cl^-^ extruder termed KCC2 [3]. During early development [Cl^-^]_i_ is maintained at high levels by active Cl^-^ accumulation via the Na^+^-K^+^-2Cl^-^-Symporter NKCC1, rendering GABA responses depolarizing and under certain conditions excitatory [4–6]. Such excitatory GABAergic responses contribute to the generation of spontaneous neuronal activity, which is essential for the correct maturation of nervous systems [7–9].

However, the Cl^−^-fluxes through GABA_A_ receptors, which underlie GABAergic currents, change [Cl^−^]_i_ and thus temporarily affect the amplitude of subsequent GABAergic responses, a process termed ionic-plasticity [3, 10, 11]. Such activity-dependent [Cl^-^]_i_ transients have been observed in various neurons [12–19]. Ionic plasticity plays an important role for physiological functions [20–22] as well as for pathophysiological processes [23, 24]. Theoretical assumptions and computational studies indicate that the amount and duration of [Cl^-^]_i_ ionic plasticity directly depends on the relation between Cl^−^ influx and the capacity of Cl^−^ extrusion systems [3, 24–28]. In addition, the size and geometrical structure of the postsynaptic compartments critically affect the magnitude, duration and dimensions of [Cl^-^]_i_ changes upon GABAergic activation [27, 29]. Further analyses also revealed that the membrane resistance, the kinetics of GABAergic responses and the stability of bicarbonate gradients affect the magnitude and duration of [Cl^-^]_i_ changes [12, 27, 30].

Another factor that directly influences the amount of GABA_A_ receptor-mediated Cl^-^ fluxes is a coincident membrane depolarization [11, 27, 31]. Accordingly, recent in-vitro and in-silico studies demonstrated that coincident glutamatergic depolarization profoundly augments the GABA_A_ receptor mediated Cl^-^ fluxes [32, 33]. However, to our knowledge it has not been analyzed how the interdependency between glutamatergic and GABAergic inputs affects the magnitude and the spatiotemporal properties of activity dependent [Cl^-^]_i_ changes.

In order to determine the influence of coincident glutamate stimulation on GABA_A_ receptor-induced [Cl^-^]_i_ transients, we utilized a computational model of [Cl^-^]_i_ homeostasis in the NEURON environment [25, 30]. Using a simple ball-and-stick geometry, we were able to show that the conductance and decay kinetics of glutamatergic responses directly affect the size of GABAergic [Cl^-^]_i_ changes, with a complex spatiotemporal dependency between glutamatergic and GABAergic inputs. Furthermore, we employed the model to uncover the contribution of coincident glutamatergic activity on the activity-dependent [Cl^-^]_i_ transients during simulated giant-depolarizing potentials (GDPs) [17, 30]. GDPs represent correlated spontaneous network events crucial for the development of neuronal circuits [34–36]. These simulations demonstrate for the first time that coincident glutamatergic stimulation with realistic parameters substantially modifies the GABA_A_ receptor-induced [Cl^-^]_i_ changes.

## Results

**Fig 1:**
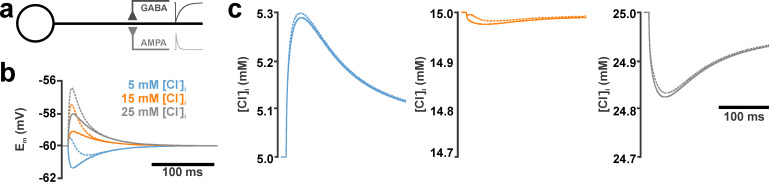
Effect of AMPA co-activation on GABA_A_ receptor mediated [Cl^-^]_i_ transients for a single GABAergic and glutamatergic input in the ball-and-stick neuronal model. (a) Schematic illustration of the compartmental model. Both GABA and AMPA synapse were located in the middle of the dendrite. The inset traces represent schematic illustrations of AMPA- and GABA-receptor mediated currents. (b) Membrane potential (E_m_) changes induced at the site of the GABAergic synapse by a single GABAergic stimulation (g_GABA_ = 0.789 nS, τ_GABA_ = 37 ms) at 5 mM (red), 15 mM (green) and 25 mM [Cl^-^]_i_ (blue). Solid lines represent the effect upon an isolated GABAergic stimulation, while dashed lines represent responses upon AMPA co-stimulation (g_AMPA_ = 0.305 nS, τ_AMPA_ = 11 ms). (c) [Cl^-^]_i_ transients induced at the site of the GABAergic synapse by the isolated GABAergic stimulation (solid lines) or GABA/AMPA co-stimulation (dashed lines). Parameters as in (b). Note that under these conditions AMPA co-activation slightly enhances the [Cl^-^]_i_ increase at 5 mM, while it slightly attenuates the [Cl^-^]_i_ decline at higher [Cl^-^]_i_ concentrations.

**Fig 2:**
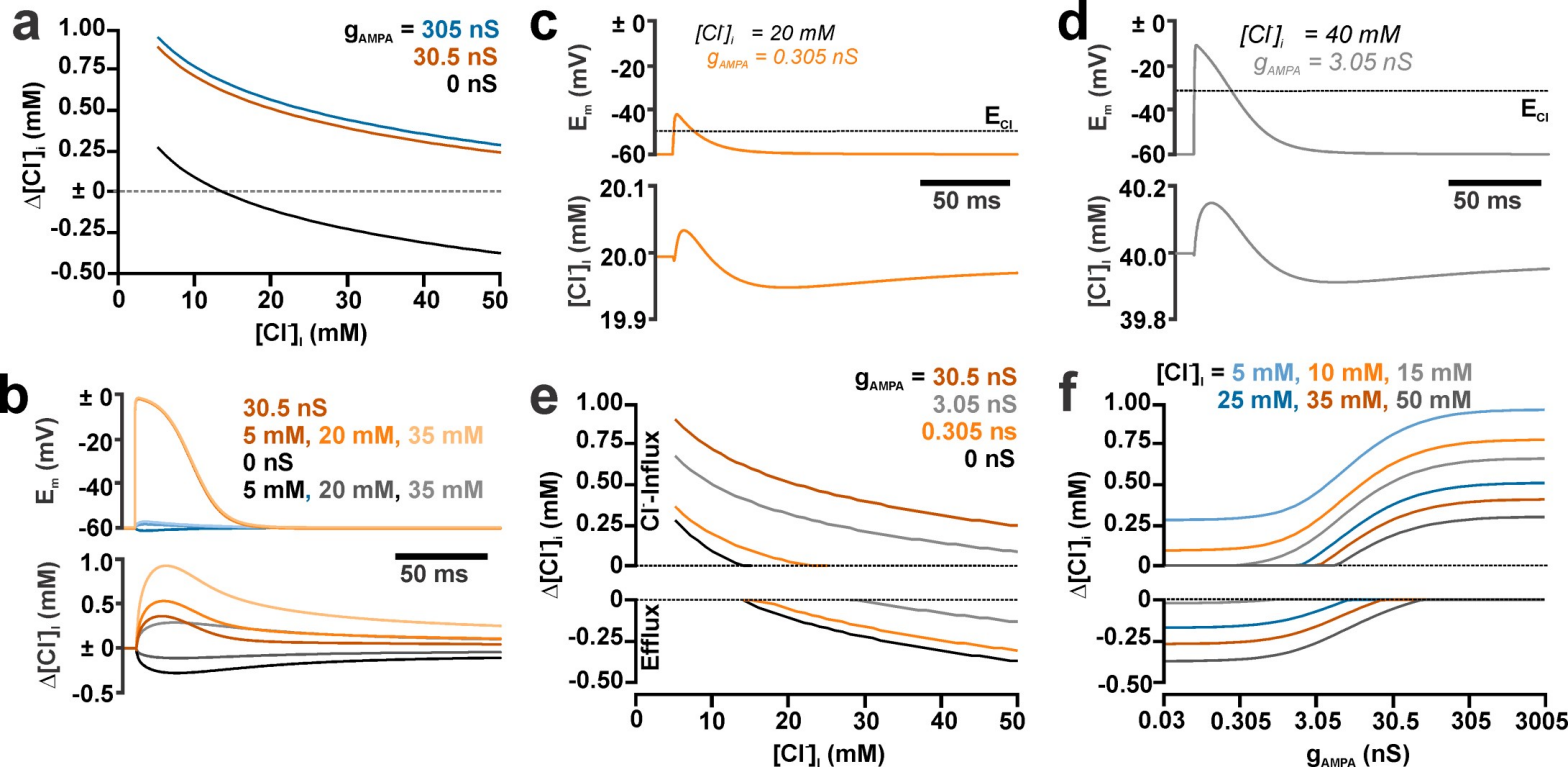
Effect of g_AMPA_ on the [Cl^-^]_i_ transients induced by GABA_A_ and AMPA receptor co-activation. (a) GABA_A_ receptor-induced [Cl^-^]_i_ changes simulated at different [Cl^-^]_i_^0^ in the absence (black trace) and the presence of AMPA receptor co-activation with high g_AMPA_ as indicated in the plot. (b) Membrane depolarization (upper panel) and [Cl^-^]_i_ (lower panel) at three different [Cl^-^]_i_^0^, as indicated in the plot, in the presence and absence of AMPA receptor co-activation. Note that this co-activation systematically shifted the [Cl^-^]_i_ transients towards higher [Cl^-^]_i_. (c) Membrane depolarization (upper panel) and [Cl^-^]_i_ (lower panel) at a [Cl^-^]_i_^0^ of 20 mM and a g_AMPA_ of 0.305 nS. Note the biphasic [Cl^-^]_i_ transient and that the Cl^-^ influx was limited to the interval when Em is above E_Cl_ (dashed line). (d) As in c but for 40 mM and g_AMPA_ of 3.05 nS. (e) Dependency of the GABA_A_ receptor-induced [Cl^-^]_i_ changes on the [Cl^-^]_i_^0^ at different g_AMPA,_ as indicated in the plot. The upper plot represents the maximal Cl^-^ influx and the lower plot the maximal Cl^-^ efflux. (f) g_AMPA_ dependency of the GABA_A_ receptors induced [Cl^-^]_i_ changes at different [Cl^-^]_i_^0^ as indicated in the plot. Note the shift towards Cl- influx with increasing g_AMPA_. In all simulations g_GABA_ = 0.789 nS, τ_GABA_ = 37 ms and τ_AMPA_ = 11 ms.

**Fig 3:**
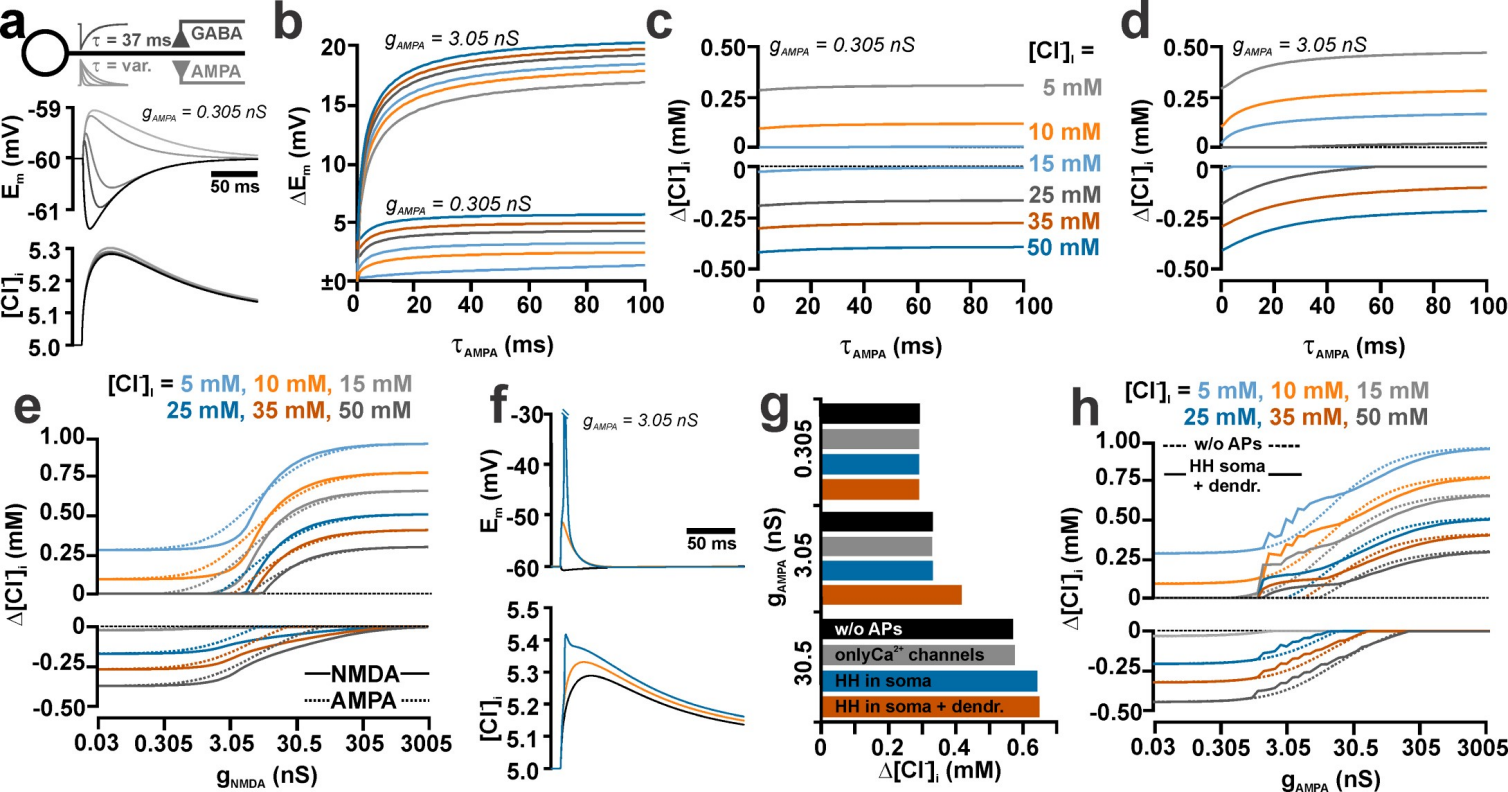
Effect of τ_AMPA_ on the [Cl^-^]_i_ transients induced by GABA_A_ and AMPA receptor co-stimulation. (a) Schematic illustration of the compartmental model. The inset traces represent schematic illustrations of AMPA- and GABA-receptor mediated currents. The GABA synapse (g_GABA_ = 0.789 nS) was stimulated with a constant τ_GABA_ of 37 ms, while τ_AMPA_ was systematically varied. The traces below the scheme displays the voltage responses (left panel) and [Cl^-^]_i_ changes induced without (black traces) or with AMPA co-stimulation (g_AMPA_ = 0.305 nS) at τ_AMPA_ values of 5, 11, 37, and 50 ms (in ascending gray shades). Note the biphasic voltage responses at short τ_AMPA_. (b) Membrane potential changes induced by a AMPA/GABA co-stimulation at different τ_AMPA_ simulated for different [Cl^-^]_i_ (for color code see panel c). The upper traces were simulated using a g_AMPA_ of 0.305 nS, while the lower traces represent simulation with g_AMPA_ of 3.05 nS. Note that the depolarization increased with larger τ_AMPA_. (c) [Cl^-^]_i_ transients induced by AMPA/GABA co-stimulation at different τ_AMPA_ simulated with different [Cl^-^]_i_ (color coded) for a g_AMPA_ of 0.305 nS. (d) As in c) but for a g_AMPA_ of 3.05 nA. Note that the [Cl^-^]_i_ changes were shifted towards diminished Cl^-^ efflux or enhanced Cl^-^ influx with larger τ_AMPA_. (e) Effect of NMDA receptor-mediated synaptic inputs with various g_NMDA_ on the [Cl^-^]_i_ transients at different [Cl^-^]_i_^0^ (color coded). The dashed traces represent the responses using AMPA receptors. Note the right shift and steeper rising phase of the NMDA responses. (f) Voltage responses and [Cl^-^]_i_ changes with only GABAergic synaptic inputs (black trace) and with simultaneous AMPA inputs in a passive dendrite (orange trace) or in a dendrite with an active HH mechanism (blue trace). (g) Effect of different active properties of the dendrite on [Cl^-^]_i_ transients at a [Cl^-^]_i_^0^ of 5 mM. Note the lack of influence of Ca^2+^ channels and that at g_AMPA_ ≥ 3.05 active dendritic properties augment the [Cl^-^]_i_ transients. (h) Effect of active dendritic properties on the [Cl^-^]_i_ transients at different [Cl^-^]_i_^0^ (color coded). The dashed traces represent the responses in the passive model.

**Fig 4:**
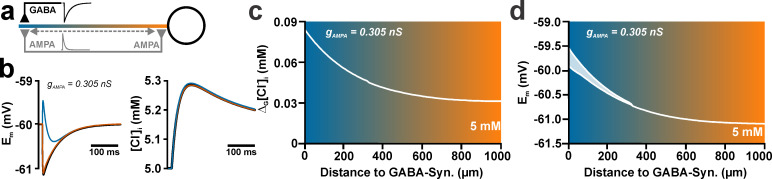
Effect of the spatial relation between individual GABA and AMPA synapses on the activity-dependent [Cl^-^]_i_ transients. (a) Schematic illustration of experimental conditions. The inset traces represent schematic illustrations of AMPA- and GABA-receptor mediated currents. (b) Voltage responses (left panel) and [Cl^-^]_i_ changes (right panel) induced without (black traces) or with AMPA co-stimulation (g_AMPA_ = 0.305 nS) with the AMPA synapse located at the site of the GABA synapse (blue) or close to the soma (orange). [Cl^-^]_i_^0^ was 5 mM in these experiments. Note that the depolarization and [Cl^-^]_i_ responses are slightly diminished if the AMPA synapse was distant to the GABA synapse. (c) Effect of the distance between AMPA and GABA synapses on the additional [Cl^-^]_i_ influx induced by AMPA co-stimulation, as compared to a GABA stimulation without AMPA (Δ_G_[Cl^-^]_i_). Note the exponential decay of Δ_G_[Cl^-^]_i_ with increasing distance between GABA and AMPA synapses. (d) Effect of the distance between AMPA and GABA synapses on the resulting membrane depolarization. The upper and lower lines represent the maximal de- and hyperpolarizing effects for biphasic responses; above 400 μm only a monophasic hyperpolarization occurred.

**Fig 5:**
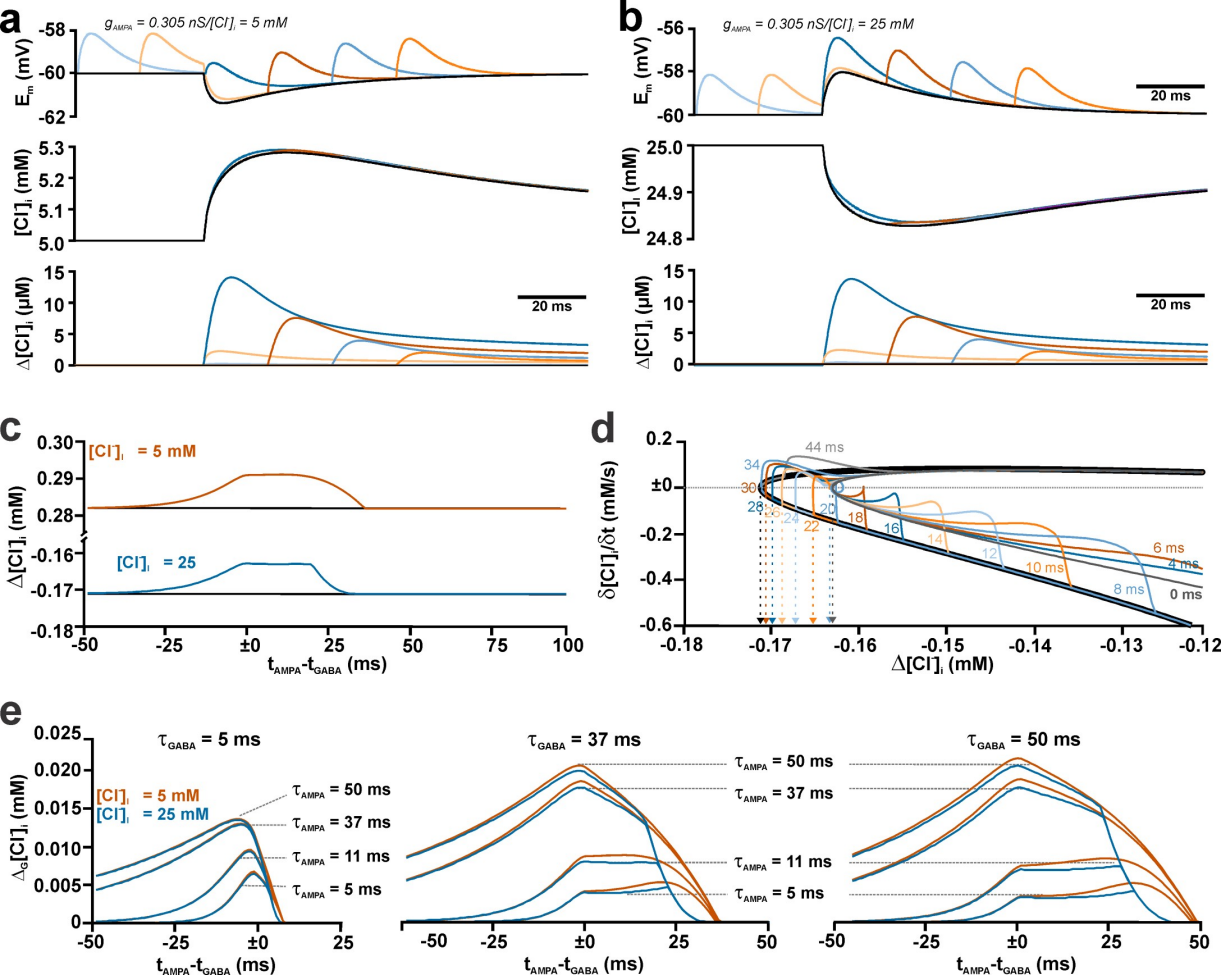
Effect of the temporal relation between single dendritic GABA and AMPA synapses on the activity-dependent [Cl^-^]_i_ transients. (a) Voltage (upper panel) and [Cl^-^]_i_ traces (lower panel) of [Cl^-^]_i_ changes induced by GABAergic stimulation without AMPA (black trace) or with AMPA stimulation at different latencies (colored traces) at a [Cl^-^]_i_^0^ of 5 mM. (b) Same as in (a) for a [Cl^-^]_i_^0^ of 25 mM. (c) Plot of the peak [Cl^-^]_i_ change at various latencies between GABA and AMPA inputs for a [Cl^-^]_i_^0^ of 5 mM (orange trace) and 25 mM (blue trace). The black lines represent the [Cl^-^]_i_ change induced by stimulation of GABA synapses only. Note the obvious “plateau”-like phases in the [Cl^-^]_i_ changes that were additionally induced by AMPA co-stimulation (Δ_G_[Cl^-^]_i_). (d) Phase plane plot of the activity-dependent [Cl^-^]_i_ transients (efflux) at a [Cl^-^]_i_^0^ of 25 mM. The black line represents the trajectory of a pure GABA stimulation. Note that all trajectories of 0 to 20 ms latency curves converged and crossed the 0 y-axis value (∂[Cl^-^]_i_ /∂t = 0 mM/s; dashed line) at a less negative x-axis value (Δ[Cl^-^]_i_) than the crossings of the >22 ms latency curves. This indicates that 0–20 ms latencies similarly and robustly decrease the peak GABA-induced efflux (i.e. 0 y-axis crossing of the pure GABA black line). See main text for further description. (e) Dependency of Δ_G_[Cl^-^]_i_ on the latency between AMPA and GABA stimulation simulated for different τ_GABA_ and τ_AMPA_. Note the plateau-like phases occurring for τ_GABA_ ≥ 37 ms at short τ_AMPA_.

**Fig 6:**
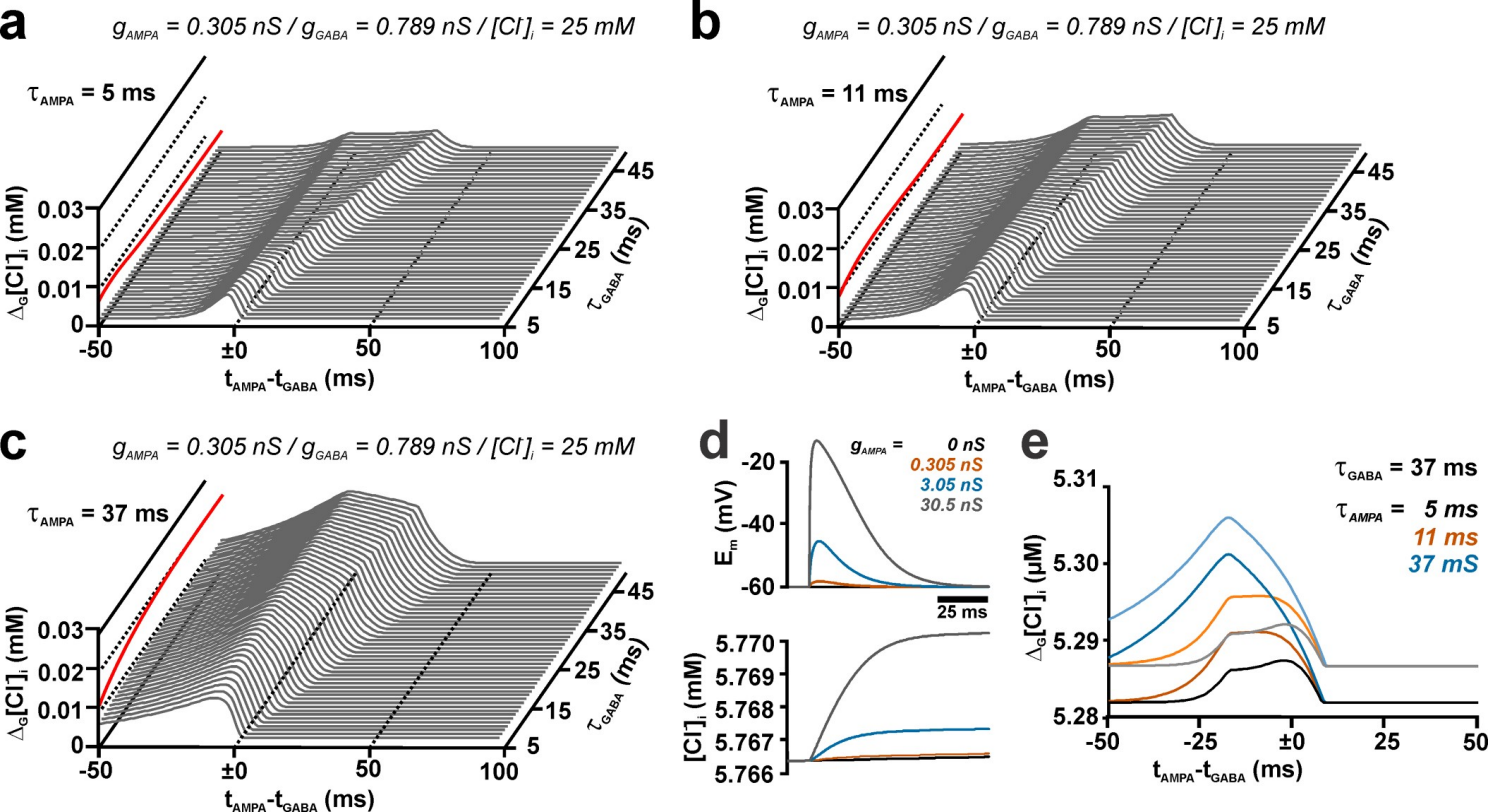
Effect of the relation between kinetics of individual dendritic GABA and AMPA synapses on the activity-dependent [Cl^-^]_i_ transients at different temporal relations between both synapses. (a-c) Amplitude of the additional [Cl^-^]_i_ influx induced by AMPA co-stimulation (compared to a GABA stimulation without AMPA) at different intervals between AMPA and GABA activation. Simulations were performed for 3 different τ_AMPA_ of 5 ms (a), 11 ms (b), and 37 ms (c) with τ_GABA_ varying between 5 ms and 50 ms. Note the evolution of a “plateau-like” phase with increasing τ_GABA_. The peak [Cl^-^]_i_ change was displayed as red trace on the left face of the plots. Note that for τ_AMPA_ of 5 ms and 11 ms, increasing τ_GABA_ above 10 ms and 17 ms reduced the [Cl^-^]_i_ shift induced by AMPA co-stimulation. (d) Voltage (upper panel) and [Cl^-^]_i_ traces induced by AMPA conductance activation (τ_AMPA_ = 11 ms) in the presence of a tonic GABAergic conductance of 8.75 nS/cm^2^. Please note the minimal [Cl^-^]_i_ changes induced under this condition. (e) Activity-dependent [Cl^-^]_i_ transients at different intervals between AMPA and GABA activation in the absence (dark colors) and presence of the tonic GABAergic conductance (light colors). Note that the amount of activity dependent [Cl^-^]_i_ changes was unaffected by the tonic current, despite a shift in the basal [Cl^-^]_i_ under these conditions.

**Fig 7:**
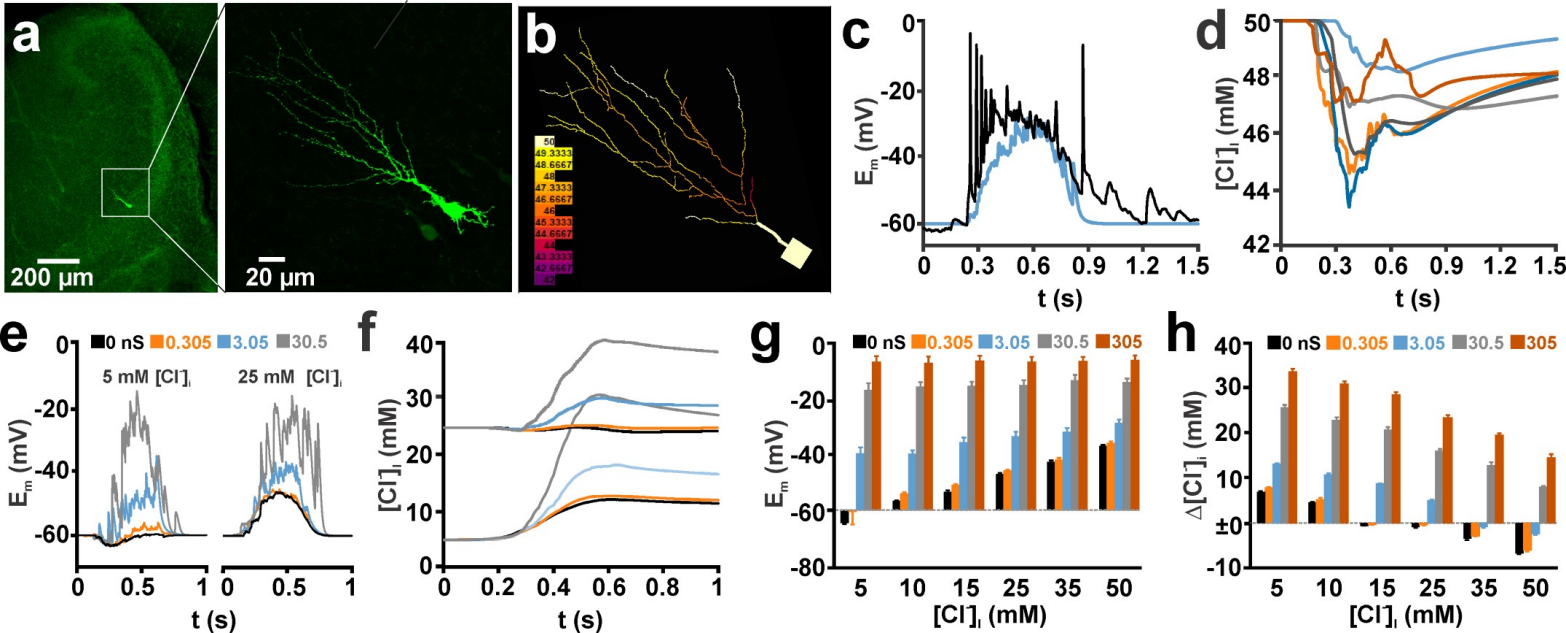
Effect of AMPA receptor co-stimulation on GABA_A_ receptor-induced [Cl^-^]_i_ transients in a morphologically realistic neuronal model stimulated with multiple spatially distributed synaptic inputs replicating GDP-like depolarizations (GDPs are experimentally observed giant depolarizing potentials in immature neurons). (a) Microfluorimetric image of a biocytin-filled CA3 pyramidal neuron stained during an electrophysiological recording. (b) Morphological representation of this neuron in the NEURON environment. The colors represent the actual [Cl^-^]_i_ during a simulated GDP. (c) Recorded (black-trace) and simulated (blue trace) voltage deflections during a GDP with GABAergic synaptic inputs only. Note that no Hodgkin-Huxley spiking mechanisms were implemented in the model. (d) Time course of [Cl^-^]_i_ in the center node of 6 typical dendrites during this simulated GDP. (e) Typical voltage deflections during a GDP at a [Cl^-^]_i_^0^ of 5 mM (left panel) and 25 mM (right panel) using different amounts of AMPA co-stimulation inputs as indicated by the color code. Note the substantial shift towards depolarized potentials at high g_AMPA_. (f) Time course of average dendritic [Cl^-^]_i_ at different [Cl^-^]_i_^0^ and g_AMPA_ (as indicated in e). Note that at a [Cl^-^]_i_^0^ of 5 mM the maximal [Cl^-^]_i_ change was augmented by addition of 107 AMPA synapses with a g_AMPA_ of 0.305 nS, while the [Cl^-^]_i_ decline at a [Cl^-^]_i_^0^ of 25 mM was attenuated by this AMPA co-stimulation. (g) Statistical analysis of the voltage changes induced by simulated GDPs with the given [Cl^-^]_i_^0^ and g_AMPA_. (h) Statistical analysis of [Cl^-^]_i_ changes induced by simulated GDPs with the given [Cl^-^]_i_^0^ and g_AMPA_. Bars represent mean ± SD of 9 repetitions. Panel (a) used with permission from Lombardi et al. [17].

**Fig 8:**
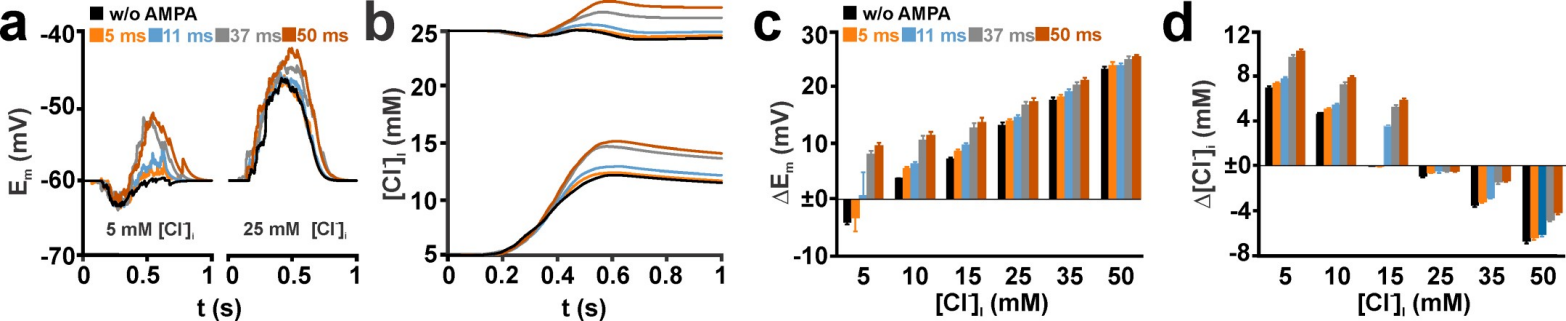
Effect of the decay time of AMPA receptor-mediated currents (τ_AMPA_) on activity-dependent [Cl^-^]_i_ transients induced by multiple spatially distributed synaptic inputs replicating GDP-like depolarizations. (a) Typical voltage traces in a simulated CA3 pyramidal neuron upon GABA stimulation (black trace) and GABA-AMPA co-stimulation with different τ_AMPA_ (color coded). Left traces represent stimulations at a [Cl^-^]_i_^0^ of 5 mM, right traces at a [Cl^-^]_i_^0^ of 25 mM. Note that a prolongation of τ_AMPA_ above 11 ms led to an obvious shift of the voltage response to positive values. (b) Average [Cl^-^]_i_ observed in these experiments. Note that [Cl^-^]_i_ changes upon GABA-AMPA co-stimulation were only slightly shifted at τ_AMPA_ of 5 and 11 ms, while at 37 ms and 50 ms an obvious shift towards Cl^-^ efflux was induced. (c) Analysis of the voltage responses upon AMPA-GABA co-stimulation. (d) Analysis of the average [Cl^-^]_i_ changes upon GABA-AMPA co-stimulation. Note that for small [Cl^-^]_i_^0^ longer τ_AMPA_ augmented the Cl^-^ -influx, while at high [Cl^-^]_i_^0^ prolongation of τ_AMPA_ diminished the Cl^-^ -efflux. Bars represent mean ± SD of 9 repetitions.

**Fig 9:**
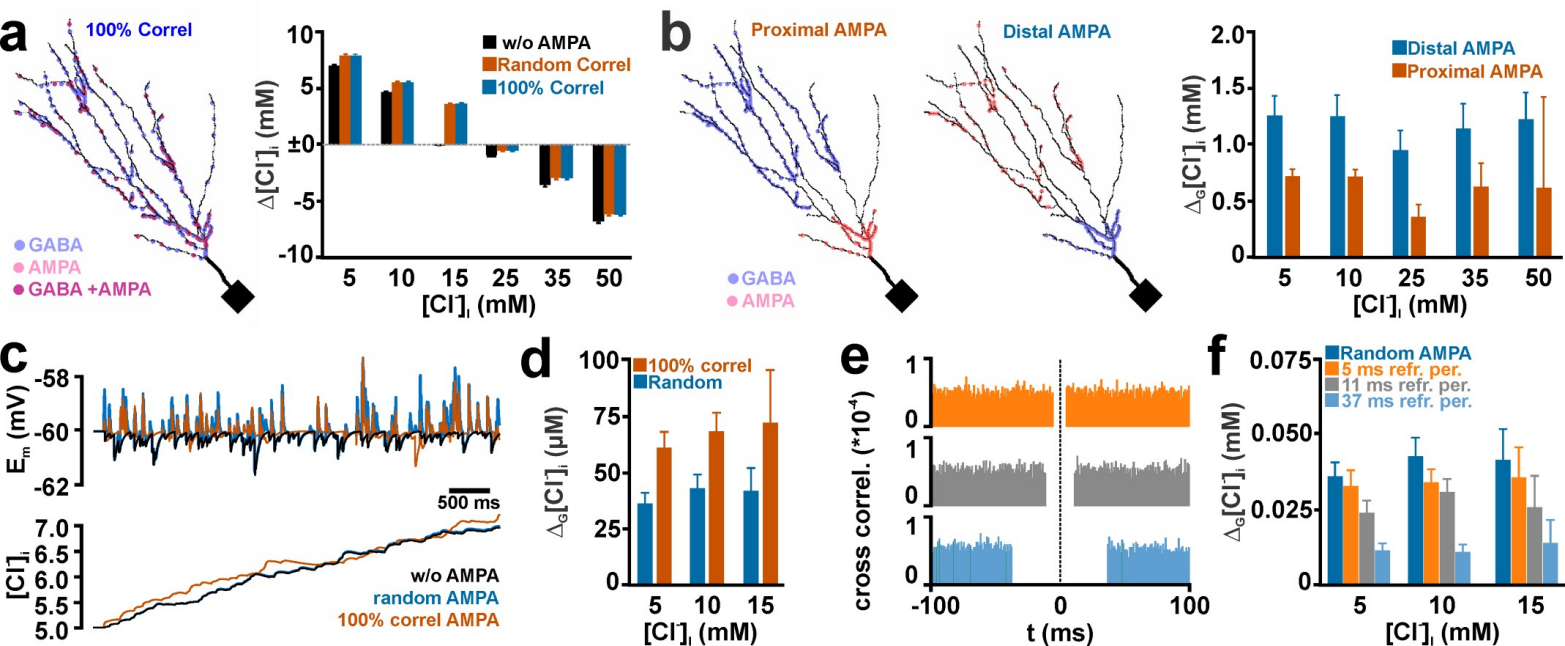
Effect of spatial and temporal correlation between multiple dendritic GABA and AMPA synapse activation on the activity-dependent [Cl^-^]_i_ transients. (a) Effect of randomly distributed (orange columns) and GABA colocalized (blue columns) AMPA inputs on GDP-induced [Cl^-^]_i_ transients. Note that both distributions had identical effects. In the left panel the positions of the synapses in the dendritic compartment are plotted. (b) In the left panels the localization of AMPA receptors in proximal and distal dendrites are displayed. Note that GABA synapses were placed in the opposite (distal and proximal) compartment. The right plot indicates that significantly larger effects of AMPA co-stimulation were observed if the AMPA synapses were positioned in the proximal compartment. (c) Typical voltage and [Cl^-^]_i_ traces observed upon random stimulation of GABA synapses without AMPA co-stimulation (black), random AMPA co-stimulation (blue), or with temporally perfectly correlated AMPA inputs (orange). (d) Statistical analysis of the additional [Cl^-^]_i_ increase upon AMPA co-stimulation (Δ_G_[Cl^-^]_i_)_._ (e) Cross correlograms of simulations for refractory period of 5 ms (orange), 11 ms (gray), and 37 ms (blue) between GABA and AMPA stimuli. The relative occurrence of AMPA stimuli in relation to each GABA stimulus was plotted, demonstrating the lack of AMPA receptor mediated synaptic input in the given refractory intervals. (f) Statistical analysis of Δ_G_[Cl^-^]_i_ at different refractory periods. Note that at refractory periods of ≥ 11 ms significantly smaller Δ_G_[Cl^-^]_i_ occurred. Bars represent mean ± SD of 9 repetitions.

In order to analyze how co-activation of depolarizing neurotransmitter receptors affects the [Cl^-^]_i_ transients evoked by GABA_A_ receptor activation, we used a previously established biophysical model of Cl^-^ dynamics in the NEURON environment [17, 25]. The model allows for realistic simulations of activity-dependent transmembrane Cl^-^ fluxes, intracellular Cl^-^ diffusion and corresponding intracellular Cl^-^ accumulation or depletion (see Methods). In a first step we computed the GABA-induced [Cl^-^]_i_ changes in a ball-and-stick model with a single dendrite (Fig 1A), in order to enable a detailed mechanistic understanding of the interaction between a single or a small group of GABA_A_ receptor-mediated and glutamate receptor-mediated synaptic inputs (Figs 1–6). Subsequently we utilized a model of a reconstructed CA3 pyramidal neuron [17, 30] stimulated by a large number of inputs to compute a more realistic estimation of how the GABA_A_ receptor-mediated [Cl^-^]_i_ transients in neurons are influenced by correlated co-activation of glutamate receptors (Figs 7–9).

### Effect of GABA and AMPA co-activation for a weak focal synaptic activation

First we analyzed the effect of glutamate receptor co-activation on the GABA_A_ receptor-induced [Cl^-^]_i_ transients. It is important to emphasize that simulations of single or a low number of synaptic inputs necessarily led to small absolute effects of glutamatergic activation on GABAergic [Cl^-^]_i_ or membrane voltage (E_m_) changes (Figs 1–6), but nevertheless allowed for systematic parameter scans important for the detailed understanding of glutamatergic effects (see below). For quantitatively stronger effects during more realistic GABAergic and glutamatergic distributed input activation see Sections 2.4 and 2.5.

Simulation of a single GABA synapse using parameters determined in-vitro for spontaneous GABAergic postsynaptic currents (PSCs) (g_GABA_ = 0.789 nS, τ_GABA_ = 37 ms) [17], induced [Cl^-^]_i_ changes (Fig 1B and 1C) that depended on the initial [Cl^-^]_i_ ([Cl^-^]_i_^0^), as has been shown before [17]. At low [Cl^-^]_i_^0^ the inwardly directed driving force (DF_GABA_ = E_m_—E_GABA_) caused a Cl^-^-influx and thus a membrane hyperpolarization. At higher [Cl^-^]_i_^0^ of 25 mM the outwardly directed DF_GABA_ caused a Cl^-^-efflux and thus a membrane depolarization, while at an [Cl^-^]_i_^0^ of 15 mM, which is close to the [Cl^-^]_i_ given by a passive distribution at -60 mV, only a slight [Cl^-^]_i_ efflux was induced (Fig 1B and 1C). The simultaneous co-activation with a simulated single glutamate synapse, using parameters that were determined in-vitro for spontaneous glutamatergic PSCs [17]) and resemble the properties of the AMPA subtype of glutamate receptors *(g*_*AMPA*_ *= 0*.*305 nS*, *τ*_*AMPA*_ *= 11 ms)*, imposed an additional depolarizing drive to the E_m_ responses (Fig 1B, dashed lines). Thereby it caused slight changes in the activity-dependent [Cl^-^]_i_ transients towards higher concentration, thus enhancing the [Cl^-^]_i_ increase at low [Cl^-^]_i_^0^ and attenuating the [Cl^-^]_i_ decrease at higher [Cl^-^]_i_^0^ (Fig 1C, dashed lines).

Since physiological and pathophysiological activity is typically characterized by neurotransmitter release from several release sites [37, 38], we next analyzed the effect of varying g_AMPA_ on the GABA_A_ receptor -mediated [Cl^-^]_i_ transient. As expected, these simulations showed that GABA_A_ receptor-induced Cl^-^ fluxes depended on the [Cl^-^]_i_^0^ (Fig 2A black trace). However, the Cl^-^ fluxes were systematically shifted towards Cl^-^ influx in the presence of AMPA receptor-mediated inputs (Fig 2A and 2B). At a g_AMPA_ of 305 nS and 30.5 nS (corresponding to 100x and 1000x glutamatergic PSCs) a consistent Cl^-^ influx was induced even at high [Cl^-^]_i_ (Fig 2A and 2B). At a g_AMPA_ of 0.305 nS (Fig 2C and 2E) and 3.05 nS (Fig 2D and 2E) bimodal effects, consisting of an initial influx followed by an efflux, were observed within a limited [Cl^-^]_i_ range. These biphasic responses were caused by the fact that the membrane depolarization only temporarily exceeds E_Cl_ (Fig 2C and 2D). The maximal values of Cl^-^ in- and efflux are displayed in separate panels in Fig 2E and 2F.

A systematic analysis of the direction of Cl^-^ fluxes at various g_AMPA_ revealed that the GABA_A_ receptor-mediated Cl^-^ fluxes show a logarithmic dependency on g_AMPA_, resulting in sigmoidal curves in a monologarithmic plot (Fig 2F). Only at low [Cl^-^]_i_ concentrations, which permit a Cl^-^ influx even without AMPA co-stimulation, a monotonous increase in the Cl- fluxes were induced by increasing g_AMPA_ (Fig 2E and 2F). When [Cl^-^]_i_ was above 13 mM (corresponding to E_Cl_ above -60 mV) addition of g_AMPA_ caused biphasic Cl- fluxes (Fig 2E and 2F).

In summary, these results indicate that co-activation of AMPA receptors enhances Cl^-^ influx at low [Cl^-^]_i_ and attenuates Cl^-^ efflux at high [Cl^-^]_i_. with the possibility to even revert the latter to Cl^-^ influx. Already relatively low, physiologically relevant g_AMPA_ values representing 10–100 spontaneous postsynaptic events, are sufficient to substantially modulate GABA_A_ receptor mediated Cl^-^ fluxes.

### Influence of τ_AMPA_ on GABA_A_ receptor-induced [Cl^-^]_i_ transients

Next we analyzed the influence of the kinetics of coincident AMPA receptor activation on the GABA_A_ receptor-induced [Cl^-^]_i_ transients. For this purpose, we modified the decay time constant of the AMPA synapse (τ_AMPA_) using values between 1 and 100 ms. These simulations were performed under consideration of the experimentally determined values for GABA_A_ receptor-mediated inputs either at g_AMPA_ of 0.305 nS (i.e. the experimentally determined value [17]) or at g_AMPA_ of 3.05 nS (to estimate the effects of a small number of coincident colocalized glutamatergic inputs). As the depolarizing effect of the AMPA synapse strictly depends on τ_AMPA_ (Fig 3A), for τ_AMPA_ values < 37 ms biphasic voltage responses were induced at low [Cl^-^]_i_ (Fig 3A). In contrast, at high [Cl^-^]_i_ (where GABAergic responses are depolarizing) the peak depolarization was enhanced. For τ_AMPA_ values > 37 ms the depolarization was virtually saturated (Fig 3B). In accordance with this observation the AMPA-mediated shift in the GABAergic [Cl^-^]_i_ transients was sensitive to τ_AMPA_ at values < 37 ms, while at values > 37ms only minimal additional effects were observed (Fig 3C and 3D). These findings indicate that the kinetics of glutamatergic synaptic inputs have a consistent effect on activity-dependent [Cl^-^]_i_ changes and that shorter glutamatergic postsynaptic potentials (PSPs) will attenuate the effect of AMPA on activity-dependent [Cl^-^]_i_ transients.

Since these results implicate a substantial influence of the time course of depolarizing events on the GABA-induced [Cl^-^]_i_ transients, we also investigated the effect of NMDA-receptors, which possess slow onset and decay kinetics, on the GABA receptor-induced [Cl^-^]_i_ transients. These simulations with an established model for the NMDA receptor [39] revealed that co-activation of NMDA receptors (τ_NMDA_ = 500 ms) induced at high g_NMDA_ [Cl^-^]_i_ transients that are similar as the ones induced by AMPA receptor co-activation (Fig 3E). However, the [Cl^-^]_i_ transients showed a steeper dependency on g_NMDA_, probably reflecting the Mg^2+^ block [40]. Thus at moderate g_NMDA_ of ~3 nS the [Cl^-^]_i_ changes were lower for a given NMDA conductance than for a similar AMPA conductance (Fig 3E).

In addition to the long-lasting NMDA receptor mediated responses, short action potentials may also provide a depolarizing drive that enhances the GABA induced [Cl^-^]_i_ transients. To investigate their influence, we implemented an established Hodgkin-Huxley (HH) mechanism [41], either only in the soma or in the soma and the dendrite, to emulate either passively or actively back-propagating actions potentials, respectively. These simulations revealed that action potentials enhanced the effect of AMPA on the GABAergic [Cl^-^]_i_ transients (Fig 3F–3H). While at a low g_AMPA_ of 0.305 nS no AP was induced and thus no additional [Cl^-^]_i_ influx was induced, at a moderate g_AMPA_ of 3.05 nS only actively back-propagating APs augment the [Cl^-^]_i_ changes (Fig 3G and 3H). At higher g_AMPA_ also somatic APs, that spread passively (electrotonically) into the dendritic compartment, were able to augment the [Cl^-^]_i_ transients (Fig 3G). The addition of voltage gated Ca^2+^ channels [41] in the dendrite had no effect on the [Cl^-^]_i_ transients (Fig 3G).

### Spatial and temporal constrains of AMPA receptor-mediated shift in GABAergic [Cl^-^]_i_ transients for a weak focal synaptic activation

To analyze how the spatial distance between the AMPA and the GABA synapse influences the GABA_A_ receptor-induced [Cl^-^]_i_ transients, we systematically moved the AMPA synapse along the dendrite from the somatic (0%) to the distal end (100%) (Fig 4A and 4B) using the experimentally determined parameters for AMPA and GABA inputs (g_GABA_ = 0.789 nS, τ_GABA_ = 37 ms, g_AMPA_ = 0.305 nS, τ_AMPA_ = 11 ms [17]). These experiments revealed that the membrane potential change depends on the location of the AMPA synapse. The maximal amplitude of the positive shift in E_m_ induced by the AMPA co-stimulation decreased substantially at distant positions (Fig 4B), due to the leak conductance shunting the synaptic currents (i.e. dendritic filtering). In accordance with these smaller depolarizing E_m_ shifts, the [Cl^-^]_i_ changes were slightly decreased with increasing distance between AMPA and GABA synapses (Fig 4B). To quantify this effect, we calculated the amount of additional [Cl^-^]_i_ changes induced by AMPA co-stimulation (Δ_G_[Cl^-^]_i_) by subtracting the [Cl^-^]_i_ changes at GABA stimulation from the [Cl^-^]_i_ changes at AMPA/GABA co-stimulation. These simulations revealed that the decrease of Δ_G_[Cl^-^]_i_ with increasing distance between both synapses could be perfectly fitted (R^2^ = 0.023) with a monoexponential function, using a decay length constant (λ) of 248 μm (Fig 4C). This is exactly the λ value determined for the attenuation of the peak voltage responses (Fig 4D) and reflects the exponential decay of voltage in a linear cable with homogeneous membrane resistance. These observations indicate that the distance between GABAergic and glutamatergic synapses is a relevant factor determining the effects of co-activation on the activity-dependent [Cl^-^]_i_ transients.

To analyze how the temporal relation between the AMPA- and the GABA-mediated activity influences the GABA_A_ receptor-induced [Cl^-^]_i_ transients we next systematically varied the delay between the AMPA and the GABAergic stimulus from -49 ms (i.e. AMPA before GABA) to +100 ms (i.e. AMPA after GABA) and determined the impact of this latency shift on the [Cl^-^]_i_ transients (Fig 5A and 5B). The parameters for AMPA and GABA inputs were identical to the parameters used before (g_GABA_ = 0.789 nS, τ_GABA_ = 37 ms, g_AMPA_ = 0.305 nS, τ_AMPA_ = 11 ms) and both synapses were located at the same position. These simulations revealed that the additional effect of AMPA co-stimulation on the [Cl^-^]_i_ transients (Δ_G_[Cl^-^]_i_) became maximal when GABA and AMPA stimulus were provided simultaneously (Fig 5C). Surprisingly, this AMPA effect on Δ_G_[Cl^-^]_i_ remained stable for a latency of ≈20 ms, before it rapidly declined (Fig 5C). To provide a mechanistic explanation for this plateau phase, we next plotted the rate of [Cl^-^]_i_ changes versus the absolute value of the [Cl^-^]_i_ change (Fig 5D). This phase plane plot illustrates that the trajectories of all AMPA stimulations with a latency between 0 and 20 ms converged with the trajectory of the 0 ms latency stimulus (purple line), thus reaching identical minimal [Cl^-^]_i_ values (obtained at the intersection with the y-axis value ∂[Cl^-^]_i_/∂t = 0 mM/s). Latencies between 22 and 34 ms provided a gradual decline in the minimal [Cl^-^]_i_. For latencies > 34 ms the additional AMPA-mediated reduction of the Cl^-^ -efflux was initiated after a minimal [Cl^-^]_i_ was reached, therefore these stimulations did not provide a [Cl^-^]_i_ decrease exceeding the [Cl^-^]_i_ decrease mediated by a pure GABA stimulation (black line).

To investigate how the duration of GABA and AMPA receptor-mediated currents influence this complex time course of [Cl^-^]_i_ transients, we next systematically varied τ_GABA_ (5 ms, 37, ms 50 ms) as well as τ_AMPA_ (5 ms, 11 ms, 37, ms 50 ms) and determined Δ_G_[Cl^-^]_i_ (Fig 5E). These simulations revealed that, in accordance with the previous results, Δ_G_[Cl^-^]_i_ increased at longer τ_AMPA_ for all three τ_GABA_ tested. While at a short τ_GABA_ of 5 ms no plateau phase was observed, such a plateau occurred for τ_GABA_ of 37 ms and 50 ms with short τ_AMPA_ values of 5 ms or 11 ms (Fig 5E). However, in contrast to our hypothesis that such a plateau phase occurred when τ_AMPA_ was shorter than τ_GABA,_ for a τ_GABA_ ≥ 37 ms and τ_AMPA_ ≥ 37 ms Δ_G_[Cl^-^]_i_ steadily declined after the maximal value was obtained at simultaneous AMPA/GABA stimulation. Another finding of these simulations that appears counter-intuitive is the fact that for short τ_AMPA_ of 5 ms and 11 ms the peak Δ_G_[Cl^-^]_i_ decreases when τ_GABA_ increased from 5 ms to 37 ms (Fig 5E).

To investigate these issues in detail, we next systematically varied τ_GABA_ between 5 and 50 ms using fixed τ_AMPA_ values of 5, 11, and 37 ms and determined Δ_G_[Cl^-^]_i_ at different AMPA/GABA latencies. In these simulations we were able to identify a complex dependency between Δ_G_[Cl^-^]_i_ and the relation between τ_GABA_ and τ_AMPA_ (Fig 6A–6C). For τ_AMPA_ of 5 and 11 ms the influence of the AMPA/GABA latency on Δ_G_[Cl^-^]_i_ changed from a “peak”-like pattern to a “plateau”-like phase, in which the maximal Δ_G_[Cl^-^]_i_ remained rather constant for a progressively longer latency interval between AMPA and GABA stimulation. This “plateau”-like phase occurred under conditions when τ_GABA_ is at more than about 3 times larger than τ_AMPA_ (Fig 6A and 6B). For τ_AMPA_ of 37 ms this “plateau”-like phase was not reached, but a phase with linearly decreasing [Cl^-^]_i_ shifts became visible (Fig 6C). In any way, the maximal AMPA-dependent [Cl^-^]_i_ shift was observed at τ_GABA_ of 11 ms, 18 ms and 47 ms for τ_AMPA_ of 5 ms, 11 ms and 37 ms, respectively (red plots in Fig 6A), reproducing the observation that prolonging τ_GABA_ can reduce Δ_G_[Cl^-^]_i_. From these results it can be concluded, first, that the effect of AMPA co-stimulation on Δ_G_[Cl^-^]_i_ depends critically on the timing between both inputs, second, that Δ_G_[Cl^-^]_i_ is maximal when τ_GABA_ is slightly larger than τ_AMPA_, and, third, that a “plateau”-like interval of stable Δ_G_[Cl^-^]_i_ occurred when τ_GABA_ is at least 3 times larger than τ_AMPA_.

Next, we investigated how the time course of E_m_ changes contributes to this complex dependency. For this purpose, we used identical stimulation parameters (g_GABA_ = 0.789 nS, τ_GABA_ = 37 ms, g_AMPA_ = 0.305 nS, τ_AMPA_ = 11 ms, [Cl^-^]_i_^0^ = 25 mM) and modified the time course of the voltage traces by changing the membrane time constant (S1 Fig). These simulations revealed that reducing the membrane time constant, which enhanced the onset kinetics of AMPA and GABA receptors, as well as the decay of AMPA receptor-mediated voltage responses, had only a minor effect on the observed [Cl^-^]_i_ changes (S1A Fig). The trajectories at different latencies converge virtually in the same manner as at physiological conditions (S1B Fig). On the other hand, a substantial prolongation of the membrane time constant slowed the kinetics of both AMPA and GABA-receptor dependent depolarizations (S1C Fig) and led to significantly different behavior of [Cl^-^]_i_ changes: The delayed GABAergic depolarization increased Δ[Cl^-^]_i_ when GABAergic input was stimulated alone (black trace). In addition, the maximum amount of Δ_G_[Cl^-^]_i_ (i.e. the difference between the 0 ms latency trajectory and the GABA only trajectory) was smaller. More importantly, the trajectories for latencies > 4 ms did no longer converge with the trajectory of simultaneous AMPA/GABA stimulation (purple line), which resulted in a steady decline of Δ_G_[Cl^-^]_i_ for all conditions in which AMPA was stimulated after the GABA input. In summary, these results indicate a complex interplay between the membrane time constant and the kinetics of AMPA- and GABA-mediated synaptic events. Only in cases in which the kinetics of the receptors dominate the time course of membrane responses, the complex, plateau-like dependency of Δ_G_[Cl^-^]_i_ on the latency between GABA and AMPA inputs was observed.

While in these models the background conductance was set by a purely passive conductance, it had been demonstrated that tonic Cl^-^ currents considerably contribute to the background conductance [42]. Therefore, we additionally implemented a tonic background conductance of 8.75 nS/cm^2^ [43] and reduced the passive conductance to 0.99125 mS/cm^2^, to maintain the input resistance of 188.2 MΩ. These simulations revealed that under this condition a constant increase in the basal [Cl^-^]_i_ occurred. In the presence of this tonic [Cl^-^]_i_ conductance activation of AMPA-mediated synapses led to [Cl^-^]_i_ changes that depended on g_AMPA_ and τ_AMPA_ (S2 Fig). However, at physiologically relevant values for AMPA receptor-mediated inputs (g_AMPA_ = 0.305 nS and τ_AMPA_ = 11 ms), only a minimal additional [Cl^-^]_i_ increase by 0.1 μM was induced, which slightly increased to 0.85 μM and 3.74 μM at gAMPA of 3.05 nS and 30.05 nS, respectively (Figs 6D and S2). Accordingly, addition of a tonic GABAergic current had no effect on the [Cl^-^]_i_ changes induced by phasic GABAergic inputs, despite a slight shift in the basal [Cl^-^]_i_ (Fig 6E).

### Influence of gAMPA and τAMPA on the GABAergic [Cl^-^]_i_ transients in a morphologically realistic neuronal model with multiple distributed synaptic inputs

To investigate in a more physiological setting how the interference between GABA_A_ and AMPA receptors influence the resulting [Cl^-^]_i_ transients, we implemented GABA and AMPA synapses in a morphologically realistic model of an immature CA3 pyramidal neuron (Fig 7A and 7B), using morphology and membrane parameters determined in in-vitro experiments [17]. In the majority of simulations, we applied experimentally estimated correlated GABAergic and glutamatergic activity recorded during giant depolarizing potentials (GDPs, [17], see Fig 7C). GDPs are highly relevant network events in the immature hippocampus that have been shown to cause substantial [Cl^-^]_i_ changes [8, 17, 35, 44]. Therefore, the use of these neurons and their activity patterns allows to simulate ionic plasticity in a morphologically and physiologically relevant setting. This computational model, which incorporates basic morphological and biophysical properties of hippocampal neurons, generated complex trajectories of [Cl^-^]_i_ within individual dendrites during a simulated GDP (Fig 7D). These [Cl^-^]_i_ changes in the individual neurites are determined by Cl^-^-fluxes via GABA_A_ receptors, lateral Cl^-^ diffusion within the dendritic compartment and transmembrane Cl^-^-transport [25, 29, 33]. For a better quantification and display, the average [Cl^-^]_i_ over all nodes of all dendrites was calculated at each simulated interval (compare e.g. Fig 7F), while E_m_ was determined in the soma.

In a first set of simulations, we investigated the impact of g_AMPA_ on GABAergic [Cl^-^]_i_ changes during a simulated GDP. For the simulation of a GDP, 534 GABAergic synapses were randomly distributed within the dendrites and activated stochastically to emulate the distribution of GABAergic PSCs during a GDP observed in-vitro [17] (see Materials and Methods for detail). In accordance with previous observations [17, 30], the massive GABAergic activity during a GDP led to substantial [Cl^-^]_i_ changes, with a [Cl^-^]_i_ increase by 3.94 ± 0.05 mM (n = 9 repetitions with random distribution/stimulation) at a low [Cl^-^]_i_^0^ of 5 mM and a [Cl^-^]_i_ decrease by -3.78 ± 0.08 mM (n = 9) at a [Cl^-^]_i_^0^ of 25 mM (black traces in Fig 7E and 7F). Adding 107 AMPA synapses with a g_AMPA_ of 0.305 nS (i.e. the experimentally determined number and conductance of AMPA inputs) led to a substantial increase in the activity-dependent [Cl^-^]_i_ transients to 4.95 ± 0.07 mM for a [Cl^-^]_i_^0^ of 5 mM and significantly attenuated the activity-dependent [Cl^-^]_i_ decrease at 25 mM to -2.88 ± 0.06 mM (Fig 7F red trace). A similar trend was also observed for other [Cl^-^]_i_^0^ concentrations. Co-stimulation with 107 AMPA inputs increased the [Cl^-^]_i_ transients at low [Cl^-^]_i_^0^ and attenuated the transients at high [Cl^-^]_i_^0^ (Fig 7H). Using a larger value for g_AMPA_ enhanced this impact on activity-dependent [Cl^-^]_i_ shift (Fig 7F). At g_AMPA_ values of 30.5 and 305 nS at all investigated [Cl^-^]_i_^0^, an increase in [Cl^-^]_i_ was induced (Fig 7H), in accordance with the depolarized E_m_ induced by these conditions (Fig 7G). In summary, these results indicate that physiological levels of AMPA co-stimulation can significantly affect the GABA_A_ receptor induced [Cl^-^]_i_ transients. In line with the results from the ball-and-stick model, implementation of the tonic GABAergic conductance of 8.75 nS/cm^2^ [43] had only a negligible effect (S3 Fig). With this tonic current, the [Cl^-^]_i_ increase at physiological g_AMPA_ values of 0.305 nS was augmented by only 0.046 mM at a [Cl^-^]_i_^0^ of 5 mM and the [Cl^-^]_i_ decrease at a [Cl^-^]_i_^0^ of 25 mM was reduced by only 0.036 mM. Even at higher g_AMPA_ values the effect of tonic GABAergic conductances remained below 0.1 mM.

In the next series of simulations, we systematically altered the decay time constant τ_AMPA_ for all 107 AMPA receptor-mediated synaptic inputs during a GDP from the experimentally determined value of 11 ms to 5, 37 and 50 ms, while using g_AMPA_ of 0.305 nS and the experimentally determined values for the GABAergic synaptic inputs. Although this slight shift in τ_AMPA_ had only a minor effect on the size of the voltage deflections (Fig 8A and 8C), it significantly modified the effect of AMPA co-stimulation on GABA_A_ receptor mediated [Cl^-^]_i_ (Fig 8B and 8D). At a [Cl^-^]_i_^0^ of 5 mM the enhancement of GDP-induced [Cl^-^]_i_ transient by AMPA (Δ_G_[Cl^-^]_i_) was reduced from 1.02 ± 0.06 mM (n = 9 repetitions) at τ_AMPA_ = 11 ms to 0.48 ± 0.06 mM at τ_AMPA_ = 5 ms (Fig 8B). Similarly, at a [Cl^-^]_i_^0^ of 25 mM, Δ_G_[Cl^-^]_i_ was diminished by shortening τ_AMPA_ from 11 ms (0.93 ± 0.1 mM) to 5 ms (0.43 ± 0.07 mM) (Fig 8B). In contrast, prolonging τ_AMPA_ to 37 or even 50 ms had a substantial impact on the time course and amount of the voltage deflections (Fig 8A). Accordingly, the activity-dependent [Cl^-^]_i_ transients were systematically shifted toward more influx or less efflux, respectively (Fig 8B–8D). In summary, these results indicate that even slight changes in τ_AMPA_ can substantially influence the impact of AMPA co-activation on GABA_A_ receptor-mediated [Cl^-^]_i_ transients.

In addition, we also evaluated the effect of NMDA receptors, which possess slow onset and decay kinetics, on the GABA receptor-induced [Cl^-^]_i_ transients. These simulations, using the same temporal and spatial pattern of glutamatergic inputs, but an established model for the NMDA receptor [39], revealed that co-activation of NMDA receptors (τ_NMDA_ = 500 ms) induced in general larger [Cl^-^]_i_ transients as compared to AMPA receptor co-activation (S4 Fig). While the effects were small at a g_NMDA_ of 0.305 nS (1.2 ± 0.1 mM vs. 0.9 ± 0.1 mM at [Cl^-^]_i_^0^ of 5 mM), substantial differences were observed at intermediate g_NMDA_ values (17.5 ± 0.7 mM vs. 6.1 ± 0.3 mM at 3.05 nS or 29.3 ± 0.8 mM vs. 18.6 ± 0.7 mM at 30.5 nS) (S4 Fig).

### Spatial and temporal constrains of AMPA-mediated infuences on GABAergic [Cl^-^]_i_ transients in a morphologically realistic neuronal model

Next we analyzed how the spatial relation between GABA and AMPA synapse activation influences the size of GDP-dependent [Cl^-^]_i_ transients. For this purpose, we first compared whether direct co-localization of each of the 107 AMPA synapses with a GABA synapse (100% spatial correlation) affects the AMPA-mediated shift in the GDP-dependent [Cl^-^]_i_ transients. These simulations revealed that the activity-dependent [Cl^-^]_i_ shifts with this 100% spatially correlated AMPA synapses were not significantly different from models with a random spatial distribution of AMPA synapses (Fig 9A). Also a restriction of AMPA synapses in either the distal 25% or the proximal 25% of the dendrite length had no substantial effect on the magnitude of activity-dependent [Cl^-^]_i_ transients (S5 Fig). We propose that this lack of an effect was caused by the fact that the overall density of GABA and AMPA synapses in each of the 56 dendrites was so high, that independent of the individual synaptic localization comparable effects on DF_GABA_ were induced. Therefore, we next relocated the GABA and AMPA synapses in distinct parts of the dendritic compartment, with either AMPA synapses located only in the most proximal 12 dendritic branches and the GABA synapses in the most distal 34 dendritic branches, or vice versa (Fig 9B). In these simulations the GABA_A_ receptor-induced [Cl^-^]_i_ shift without AMPA co-stimulation differ from the previous stimulations, as the Cl^-^-influx was restricted to a subset of dendrites (Fig 9B). Under this strict regime, the location of the AMPA synapses had a slight effect on Δ_G_[Cl^-^]_i_. With all GABA synapses in the proximal dendrites and the AMPA synapses located in the distal dendrites a co-stimulation induced a Δ_G_[Cl^-^]_i_ of 1.25 ± 0.17 mM (n = 9 repetitions) at a [Cl^-^]_i_^0^ of 5 mM (Fig 9B). In contrast, when all GABA synapses were located in the distal dendrites and all AMPA synapses in the proximal dendrites co-stimulation induced a Δ_G_[Cl^-^]_i_ of 0.72 ± 0.07 mM (Fig 9B). A similar tendency was also observed for other [Cl^-^]_i_^0^ (Fig 9B). In summary, these results indicate that for a massive stimulation, like the modeled GDP, changes in the spatial correlation between AMPA and GABA receptors have only subtle effects on activity-dependent [Cl^-^]_i_ transients.

Finally, we investigated the effect of the temporal correlation between AMPA and GABA synaptic inputs on the activity-dependent [Cl^-^]_i_ changes. Since it was not possible to generate a non-trivial decorrelation of AMPA and GABA synaptic inputs during GDP-like activity, we stimulated 100 AMPA (g_AMPA_ = 0.305 nS/τ_AMPA_ = 11 ms) and 100 GABA (g_AMPA_ = 0.789 pS/τ_AMPA_ = 37 ms) synaptic inputs at a frequency of 20 Hz, using a random temporal distribution for both types of synapses. While at a [Cl^-^]_i_^0^ of 5 mM the random stimulation of only GABA synapses induced a [Cl^-^]_i_ increase by 2.06 ± 0.16 mM (n = 9 repetitions), this increase was augmented by 0.04 ± 0.005 mM upon random co-stimulation of AMPA synapses (Fig 9C and 9D. Comparable Δ_G_[Cl^-^]_i_ values were also observed for [Cl^-^]_i_^0^ of 10 mM and 15 mM. For higher [Cl^-^]_i_^0^ the trend towards larger [Cl^-^]_i_ at AMPA co-stimulation maintained. However, the large variance in the individual maximal [Cl^-^]_i_ responses in each simulation and the resulting high SD values for Δ_G_[Cl^-^]_i_ values caused that these changes were not significant. If in this stimulation paradigm the AMPA stimuli occurred exactly at the same time points as the GABAergic inputs (i.e. 100% temporal correlation), Δ_G_[Cl^-^]_i_ was significantly increased to 0.065 ± 0.008 mM (at a [Cl^-^]_i_^0^ of 5 mM), to 0.078 ± 0.009 mM (at 10 mM [Cl^-^]_i_^0^), and to 0.084 ± 0.034 mM (at 15 mM [Cl^-^]_i_^0^) (Fig 9D).

To investigate the effect of a decorrelation between GABA and AMPA inputs, we implemented an algorithm that generates refractory periods of 5, 11, and 37 ms around each GABAergic stimulus in which AMPA inputs are omitted (Fig 9E). These experiments revealed that for a [Cl^-^]_i_^0^ of 5 mM the Δ_G_[Cl^-^]_i_ values are significantly decreased from 0.041 ± 0.005 mM (n = 9 repetitions at random correlation) to 0.027 ± 0.004 at a refractory period of 11 ms, and to 0.014 ± 0.003 at a refractory period of 37 ms (Fig 9F). At a refractory period of 5 ms Δ_G_[Cl^-^]_i_ decreased only slightly and not significantly to 0.037 ± 0.006. A comparable reduction was also observed for a [Cl^-^]_i_^0^ of 10 mM (Fig 9F). At 15 mM only for a refractory period of 37 ms a significant reduction in Δ_G_[Cl^-^]_i_ was observed (from 0.048 ± 0.026 mM to 0.016 ± 0.011 mM). In summary, these results support the finding that the temporal correlation between GABA and AMPA synapses enhance activity-dependent [Cl^-^]_i_ shifts, in particular if AMPA and GABA inputs are correlated within the time range of their decay time constants.

## Discussion

Recent studies provide increasing evidence that GABAergic responses show an activity- and compartment-dependent behavior due to ionic plasticity in [Cl^-^]_i_ [11, 24]. Here we used biophysical modeling to study how coincident glutamatergic inputs enhance the [Cl^-^]_i_ changes induced by GABAergic activation. The main findings of this computational study can be summarized as follows: 1.) Glutamatergic co-stimulation had a direct effect on the GABAergic [Cl^-^]_i_ changes, thereby enhancing Cl^-^ influx at low [Cl^-^]_i_^0^ and attenuating or even reversing the Cl^-^ efflux caused at high [Cl^-^]_i_^0^. 2.) Massive glutamatergic co-stimulation promotes Cl^-^ influx at all [Cl^-^]_i_^0^, whereas physiological levels of glutamatergic co-stimulation mediate biphasic [Cl^-^]_i_ changes at intermediate [Cl^-^]_i_ levels typical for immature neurons. 3.) Keeping the decay kinetics of glutamatergic inputs below that of GABAergic inputs attenuated the [Cl^-^]_i_ changes. 4.) The spatial and temporal correlation between glutamatergic and GABAergic inputs has a substantial influence on the [Cl^-^]_i_ changes, with a surprisingly large temporal interval with correlation-independent [Cl^-^]_i_ changes. 5.) In a morphologically realistic model with physiologically relevant synaptic activity that replicates GDPs, the conductance and kinetics of correlated glutamatergic activity have a substantial impact on the [Cl^-^]_i_ changes. 6.) While the spatial correlation between distributed GABA and glutamatergic synapses has only a minor effect on activity-dependent [Cl^-^]_i_ changes, their temporal correlation has strong effects. 7.) Besides AMPA receptors also NMDA receptors and dendritic action potentials can enhance the GABAergic [Cl^-^]_i_ changes.

In the present model we emulated the membrane transport processes by two exponentially decaying functions [25, 29]. This model offers the advantage that it is computationally less demanding than other approaches, which model the [Cl^-^]_i_ dynamics by a set of transmembrane ion transporters that influence [Cl^-^]_i_ [27, 45]. On the other hand, our model does not consider additional effects that can be mediated by the influence of the glutamatergic Na^+^ and K^+^ fluxes on the NKCC1 and KCC2-based [Cl^-^]_i_ homeostasis [46]. While these oppositely directed Na^+^ and K^+^ fluxes will lead to balanced effects in the NKCC1-based [Cl^-^]_i_ homeostasis typical for immature neurons, it will attenuate the driving force for Cl^-^ extrusion in the mature KCC2-based [Cl^-^]_i_ homeostasis. Presumably, larger [Cl^-^]_i_ increases can be expected when more realistic transport processes are considered for the [Cl^-^]_i_ models. In addition, we used a purely diffusional model without considering electrodiffusion [26, 27], as it has been demonstrated recently that for Cl^-^ ions the diffusional movement is considerably larger than the contribution of electric drift [46].

In this study we investigated the effect of co-activation of glutamatergic AMPA receptors on GABA receptor-dependent [Cl^-^]_i_ changes over a wide range of [Cl^-^]_i_^0^, which gave us the opportunity to evaluate the role of ionic plasticity for mature and developing nervous systems [3, 47]. In the mature brain GABAergic activity causes an increase in [Cl^-^]_i_ [11, 16, 25, 48, 49], thereby decreasing the inhibitory action of the GABAergic system [13, 26, 27, 33]. Note in this respect that inhibition, as defined by a decrease in spike probability, is not necessarily related to a hyperpolarization, but that shunting inhibition considerably contributes to inhibition [1, 42, 50]. The present study demonstrates that glutamatergic co-stimulation enhances the GABAergic Cl^-^-influx and thus enhances the activity-dependent [Cl^-^]_i_ increase, as has recently been shown in-vitro [32] and in-silico [33]. As a consequence, the inhibitory effect of GABA may be even more impaired upon a co-activation of glutamate receptors [33]. The high basal [Cl^-^]_i_ in immature neurons leads to GABAergic Cl^-^-efflux and thus to a decrease in [Cl^-^]_i_ upon GABAergic activation [18, 51]. This [Cl^-^]_i_ decline can be associated with a loss of excitatory action and/or an enhancement of the shunting inhibitory effect of GABA receptors [21, 50, 52]. The present study demonstrates for the first time that in immature neurons a co-activation of GABA and glutamate receptors attenuates the activity-dependent [Cl^-^]_i_ decrease, thereby stabilizing the depolarizing actions of GABA.

The absolute values of the additional [Cl^-^]_i_ changes induced by AMPA co-stimulation in our ball-and-stick simulations are small, however it has to be taken into account that these changes represent the [Cl^-^]_i_ changes induced at a single synapse with a single synaptic stimulus. Physiological levels of synaptic activity may thus cause significantly larger [Cl^-^]_i_ changes, as has been shown in-vitro [32]. Even physiological levels of glutamatergic co-stimulation (0.305–3.05 nS, representing 1-10x spontaneous synaptic inputs) mediate in our model a reliable increase in Cl^-^ influx at low [Cl^-^]_i_^0^, implicating that glutamatergic co-stimulation will enhance ionic plasticity in mature neurons. Thereby coincident glutamatergic activity provides an additional challenge for the [Cl^-^]_i_ homeostasis in mature neurons and can substantially contribute to an activity-dependent decline in the inhibitory action of GABAergic synapses [33, 53, 54]. But even small Cl^-^ changes can cause substantial functional alterations for neuronal computation, as has been recently demonstrated in-silico [45, 55]. Subtle changes in GABAergic membrane responses can directly interfere with the action potential threshold [56], but may also affect the temporal fidelity of inhibitory synaptic inputs, as with a weakening of GABAergic inhibition the temporal window for effective inhibition of excitatory inputs became smaller [52].

In addition, we were also able to demonstrate that several other depolarizing events can augment the GABAergic [Cl^-^]_i_ changes. Co-activation of NMDA receptors augment the [Cl^-^]_i_ changes with a steeper dependency on the membrane conductance as compared to AMPA receptors. This behavior is probably reflecting the Mg^2+^ block of NMDA receptors [40], leading to smaller inward currents as long as E_m_ was below the threshold for the release of the Mg^2+^ block. Accordingly, at moderate conductances NMDA receptors mediate smaller effects on the [Cl^-^]_i_ changes than AMPA receptors. But under physiological conditions, with AMPA-NMDA receptor co-activation, we assume that NMDA receptors will provide an additional drive for larger [Cl^-^]_i_ changes. Accordingly, we observed in the reconstructed neurons that the simulated GDP-like activity generated larger [Cl^-^]_i_ changes when the AMPA-receptors were replaced with NMDA-receptors, because under this condition the dendritic depolarizations were sufficient to effectively remove the Mg^2+^ block [40]. In addition, we were able to demonstrate that action potential firing, generated by a Hodgkin-Huxley-like mechanism, mediates an additional increase in the [Cl^-^]_i_ changes. This suggests that under physiological conditions burst firing can substantially contribute to ionic plasticity. In contrast, the addition of a tonic GABAergic conductance had only a negligible effect of the activity-dependent [Cl^-^]_i_ changes.

As mentioned before, the high [Cl^-^]_i_ in immature neurons and the resulting GABAergic Cl^-^-efflux results in a condition in which glutamatergic co-stimulation attenuates GABAergic [Cl^-^]_i_ loss and thereby stabilizes depolarizing GABAergic actions. In addition, our computational model demonstrates that glutamatergic co-stimulation at physiologically relevant levels (g_AMPA_ of 0.305 to 3.05 nS, corresponding to 1 to 10x of experimentally determined conductance for spontaneous AMPA receptor mediated inputs) causes biphasic [Cl^-^]_i_ changes at [Cl^-^]_i_ values between 15 and 35 mM (i.e. in the typical range determined in immature neurons [4, 57, 58]). These biphasic [Cl^-^]_i_ changes rely on the fact that the activation of glutamatergic receptors transiently pushes the membrane potential above E_Cl_ and thus supports a Cl^-^-influx during this interval. The biphasic [Cl^-^]_i_ changes reduce the maximal [Cl^-^]_i_ decline at the end of the GABAergic postsynaptic potential. By this process the activity-dependent [Cl^-^]_i_ decline as well as the burden of glutamatergic co-stimulation on [Cl^-^]_i_ homeostasis is reduced. It is tempting to speculate that this limitation of activity-dependent Cl^-^-fluxes may be one reason for the inefficient Cl^-^-transport in the immature CNS [4, 22]. The functional implications of activity dependent [Cl^-^]_i_ changes in immature neurons are harder to predict, as depolarizing GABAergic responses can mediate inhibitory [59, 60] as well as excitatory actions [61–63]. However, it can be estimated that an attenuation of the GABAergic [Cl^-^]_i_ depletion will prevent/ameliorate a loss of GABAergic excitation and will prevent/ameliorate an increase in the inhibitory effect of depolarizing GABAergic responses. Thereby coincident glutamatergic activity, which is a typical feature of the correlated network events in developing neuronal systems [36, 37], can stabilize GABAergic functions.

Our simulation demonstrated that τ_AMPA_ has, as expected, a substantial influence on Δ_G_[Cl^-^]_i_, with faster AMPA events reducing the amount of ionic plasticity. Thus the shorter AMPA receptor-mediated responses at adult synapses [64, 65] can reduce the impact of AMPA/GABA co-activation on [Cl^-^]_i_ homeostasis, in addition to making transmission more precise. In this respect, it is also tempting to speculate that conditions which permit the unblocking of NMDA-receptors (a subtype of glutamate receptors that is characterized by a rather long decay and that plays an essential role in learning, [66]), will also lead to more massive burden on [Cl^-^]_i_ homeostasis. The resulting short-term decline in GABAergic inhibition will make neurons more prone to the induction of long-term potentiation.

In addition to this expected influence of τ_AMPA_ on ionic plasticity, our simulations revealed a complex dependency of Δ_G_[Cl^-^]_i_ on τ_GABA_ and τ_AMPA_. Interestingly the maximal Δ_G_[Cl^-^]_i_ values were obtained when τ_GABA_ was slightly larger than τ_AMPA_. An additional prolongation of τ_GABA_ even reduced Δ_G_[Cl^-^]_i_ and thus diminished ionic plasticity. Intriguingly, such a prolongation of τ_GABA_ led to the appearance of a “plateau”-like phase, in which Δ_G_[Cl^-^]_i_ is insensitive to the latency between GABA and AMPA inputs. The analysis of the Cl^-^-fluxes in the phase diagram revealed that this “plateau”-like phase was due to the fact, that the Cl^-^-fluxes converged over a wide range of AMPA/GABA latencies in the trajectory of [Cl^-^]_i_ changes obtained by synchronous GABAergic and glutamatergic stimulation. For short τ_AMPA_ no “plateau”-like phase could be observed because under these conditions the time course of E_m_ was mainly determined by the membrane time constant. This explanation was supported by our observation that an artificial increase in the membrane time constant by an augmented C_m_ caused a dissipation in the phase plane plot from two attractors towards more dissociated trajectories. For larger τ_AMPA_ of 37 and 50 ms such a “plateau”-line phase was not reached because at this longer time constant the decay of the voltage deflection is determined by both the inactivation of AMPA- and GABA_A_ receptors and thus a gradual decrease in DF_GABA_ occurred during the course of co-activation.

It has been suggested that under mature conditions ionic plasticity of [Cl^-^]_i_ can act as a coincidence detector for simultaneous GABAergic and glutamatergic synaptic inputs [32]. Via such a coincidence detection GABAergic inhibition will be particularly attenuated by coincident glutamatergic inputs. The resulting [Cl^-^]_i_ increase and the corresponding reduction in the inhibitory GABAergic capacity will subsequently promote the relay of excitation in neuronal networks. The intriguing finding of the present manuscript, that the effect of glutamatergic co-stimulation on the GABAergic [Cl^-^]_i_ increase was stable for a considerable latency interval between GABAergic and glutamatergic inputs would allow the system to use a stable mechanism for adjusting the gating of excitatory information. In this respect, the striking asymmetry in Δ_G_[Cl^-^]_i_ under physiological conditions of the decay times (τ_AMPA_ < τ_GABA_) implies a spike-time dependency of ionic plasticity. While glutamatergic inputs preceding GABAergic inputs have only a small effect on Δ_G_[Cl^-^]_i_, a stable shift in Δ_G_[Cl^-^]_i_ was induced when glutamatergic synapses were activated during the decay phase of GABAergic responses. A possible functional implication of this spike-time dependency would be that common feed-forward inhibitory circuits, in which synaptic inhibition always occurs after the glutamatergic inputs, will be only minimally affected by ionic plasticity, guaranteeing an efficient and stable feed-forward inhibition. Only in cases in which glutamatergic excitation was induced during ongoing GABAergic stimulation, a substantial ionic plasticity would operate. By this mechanism the inhibitory capacity of GABA will be reduced particularly in the postsynaptic neurons that receive frequent glutamatergic inputs, thereby facilitating particularly these pathways.

In summary, our results demonstrate that glutamatergic co-activation has a prominent time- and space-dependent effect on the amount of GABA-receptor mediated [Cl^-^]_i_ changes. This ionic plasticity depends on the properties of glutamatergic inputs and their temporal correlation with the GABAergic inputs. These glutamatergic modulations of GABAergic ionic plasticity could possibly contribute to short-term memory and are likely to influence information processing in the developing and mature nervous system.

## Materials and methods

### Compartmental modeling

The biophysics-based compartmental modeling was performed using the NEURON environment (neuron.yale.edu). The source code of models and stimulation files used in the present paper can be found in ModelDB (http://modeldb.yale.edu/266823). For compartmental modelling we used in the first simulations a simple ball-and-stick model (soma with d = 20 μm, linear dendrite with l = 200 μm, diameter 1 μm, and 103 nodes; *cell_soma_dendrite*.*hoc*). In the experiment investigating the role of spatial orientation between GABA and AMPA synapses, the length of the dendrite was increased to 1000 μm (*cell_soma_dendrite_long*.*hoc*). In further experiments we used a reconstructed CA3 pyramidal cell (*Cell1_Cl-HCO3_Pas*.*hoc*; [17]). We used the reconstruction of an immature CA3 pyramidal neuron for this purpose, because coincident GABAergic and glutamatergic activity used for modelling of ionic plasticity was recorded in exactly this neuron population [17]. This reconstructed neuron contained a soma (d = 15 μm), a dendritic trunk (d = 2 μm, l = 32 μm, 9 segments) and 56 dendrites (d = 0.36 μm, 9 segments each). In all of these compartments a specific axial resistance (R_a_) of 34.5 Ωcm and a specific membrane capacitance (C_m_) of 1 μFcm^-2^ were implemented. C_m_ was varied in two experiments to accelerate/decelerate the membrane time constant. A specific membrane conductance (g_*pas*_) of 1.0 mS/cm^2^ with a reversal potential of -60 mV was inserted in all neuronal elements. In some experiments we additionally implemented a tonic background conductance of 8.75 μS/cm^2^ [43] and reduced the passive conductance to 0.99125 mS/cm^2^, to maintain the input resistance of 188.2 MΩ.

GABA_A_ synapses were simulated as a postsynaptic parallel Cl^−^ and HCO_3_^−^ conductance with exponential rise and exponential decay:

$$I_{GABA}=I_{Cl}+I_{HCO3}=1/\left(1+P\right)\cdot g_{GABA}\cdot (V-E_{Cl})+P/\left(1+P\right)\cdot g_{GABA}\cdot (V-E_{HCO3})$$

where P is a fractional ionic conductance that was used to split the GABA_A_ conductance (g_GABA_) into Cl^−^ and HCO_3_^−^ conductance. E_Cl_ and E_HCO3_ were calculated from the Nernst equation. The GABA_A_ conductance was modeled using a two-term exponential function, using separate values of rise time (0.5 ms) and decay time (variable, mostly 37 ms). Parameters used in our simulations were as follows: [Cl^−^]_o_ = 133.5 mM, [HCO3^−^]_i_ = 14.1 mM, [HCO3^−^]_o_ = 24 mM, temperature = 31°C, P = 0.18. The conductance values for GABA synapses varied as given in the main text. AMPA synapses were modeled by an Exp2Syn point process using a reversal potential of 0 mV, a tau 1 value of 0.1 ms and a tau2 value of 11 ms, in accordance with the experimentally determined value [17], except where noted. The conductance values for AMPA synapses varied as given in the main text.

For the ball-and-stick model a single GABA_A_ synapse was placed in the middle of the dendrite, except where noted. The AMPA synapse was placed as indicated in the figure legends. For the simulation of a GDP in the reconstructed CA3 neuron 534 GABAergic synapses and 107 AMPA synapses were randomly distributed within the dendrites of the reconstructed neuron, except where noted. The number of GABA and AMPA synapses replicate the estimated synapse numbers during experimentally recorded GDPs [17]. The properties of these synapses were given in the results part and/or the corresponding figure legends. Nine repetitions of these simulations were generated by altering the seed values for the random functions, which resulted in a redistribution of all synapses within the dendritic compartment and the timing of their activation. The results of these repeated simulations were given and displayed as mean ± SD.

For each synapse, the index of the dendrite as well as the position within this dendrite was randomly determined using a normal distribution. The time points of GABA and AMPA inputs were determined stochastically using a normal distribution (μ = 600 ms, σ = 9000 ms for GABA and μ = 650 ms, σ = 8500 ms for AMPA), which emulates the distribution of GABAergic and glutamatergic PSCs during a GDP observed in immature hippocampal CA3 pyramidal neurons [17]. In a few simulated experiments, the 107 AMPA inputs were stimulated synchronously with a random subgroup of the GABA inputs. Since it was not possible to generate a non-trivial decorrelation of AMPA and GABA synaptic inputs during GDP-like activity, for the decorrelation experiments, we stimulated 100 AMPA and 100 GABA synaptic inputs at a frequency of 20 Hz, using a random temporal distribution for both synapses. For the decorrelation, we used a simple algorithm that generated new random numbers until the time point of an AMPA input was temporally separated by more than the given refractory periods with respect to the time points of all GABA inputs.

For analyzing the effect of NMDA receptors, we included an established model for the NMDA receptor (Exp2NMDA2 from Senselab Model DB ID: 145836 [39]) with an onset time constant of 8.8 ms and a τ_NMDA_ of 500 ms, using similar conductance values for g_NMDA_ as for the AMPA receptors. In addition, in a limited set of simulated experiments we inserted an established Hodgkin-Huxley based model of hippocampal action potentials (Model DB ID: 3263 [41]) into soma and dendrite of the ball-and-stick model to emulate somatic and back-propagating dendritic action potentials. This Hodgkin-Huxley-based model also includes a voltage-gated Ca^2+^ conductance, which we also implemented separately in soma and dendrite of the ball-and-stick model to investigate the contribution of Ca^2+^ conductances to [Cl^-^]_i_ transients.

For the modeling of the GABA_A_ receptor-induced [Cl^-^]_i_ and [HCO_3_^-^]_i_ changes, we calculated ion diffusion and uptake by standard compartmental diffusion modeling. In order to keep the computational complexity at reasonable levels, we used a purely diffusional model without considering electrodiffusion [26, 27], as recently it has been demonstrated that for Cl^-^ ions the diffusional movement is considerably larger than the contribution of electric drift [46]. To simulate intracellular Cl^-^ and HCO_3_^-^ dynamics, we adapted our previously published model [25, 30]. Longitudinal Cl^-^ diffusion along dendrites was modeled as the exchange of anions between adjacent compartments. For radial diffusion, the volume was discretized into a series of four concentric shells around a cylindrical core and Cl^-^ or HCO_3_^-^ was allowed to flow between adjacent shells. The free diffusion coefficient for Cl^-^ inside neurons was set to 2 μm^2^/ms [14] and for HCO_3_^-^ to 1.18 μm^2^/ms [67]. To simulate transmembrane transport of Cl^-^ and HCO_3_^-^, we implemented an exponential relaxation process for [Cl^-^]_i_ and [HCO_3_^-^]_i_ to resting levels [Cl^−^]_i_^rest^ or [HCO_3_^−^]_i_^rest^ with a time constant τ_Ion_.

$$\frac{d{\left[{Ion}^{-}\right]}_{i}}{dt}=\frac{{\left[{Ion}^{-}\right]}_{i}^{rest}-{\left[{Ion}^{-}\right]}_{i}}{\tau_{Ion}}$$

Cl^−^ transport was in most experiments (if not otherwise noted) modeled as bimodal process, for [Cl^-^]_i_ < [Cl^-^]_i_^rest^ τ_Ion_ was set to 174 s to emulate an NKCC1-like Cl^−^ transport mechanism. For [Cl^-^]_i_ > [Cl^-^]_i_^rest^ τ_Ion_ was set to 321 s to emulate passive Cl^-^ efflux [43].

The impact of GABAergic Cl^−^ currents on [Cl^−^]_i_ and [HCO_3_^−^]_i_ was calculated as:

$$\frac{d{\left[{Ion}^{-}\right]}_{i}}{dt}=\frac{1}{F}\frac{I_{Ion}}{volume}$$

To simulate the GABAergic activity during a GDP, a unitary peak conductance of 0.789 nS and a decay of 37 ms were applied to each GABAergic synapse, in accordance with properties of spontaneous GABAergic postsynaptic currents in CA3 pyramidal neurons [17].

For the analysis and display of the data in the ball-and-stick model we used E_m_ and [Cl^-^]_i_ values at the site of the GABAergic synapses. For the reconstructed CA3 neuron we used the somatic E_m_ and the mean [Cl^-^]_i_ within the dendritic compartment. The mean [Cl^-^]_i_ of all dendrites was calculated by averaging the [Cl^-^]_i_ at 50% of dendritic length for all 56 dendrites. This procedure mimics the experimental procedure of Lombardi et al. [17], who determined E_GABA_ by focal application in the dendritic compartment.

For the calculation of Δ[Cl^-^]_i_, the maximal deviation of [Cl^-^]_i_ upon a GABAergic stimulus ([Cl^-^]_i_^S^) was subtracted from the resting [Cl^-^]_i_ before the stimulus ([Cl^-^]_i_^R^). For biphasic responses both minimal and maximal [Cl^-^]_i_^R^ were determined and displayed. In some cases, the manifest Δ[Cl^-^]_i_ for these responses was calculated as:

$$\Delta {\left[{Cl}^{-}\right]}_{i}={\left[{Cl}^{-}\right]}_{i}^{S,min}-{\left[{Cl}^{-}\right]}_{i}^{R}\;if\;abs({\left[{Cl}^{-}\right]}_{i}^{S,min})>abs({\left[{Cl}^{-}\right]}_{i}^{S,max})$$

$$\Delta {\left[{Cl}^{-}\right]}_{i}={\left[{Cl}^{-}\right]}_{i}^{S,max}-{\left[{Cl}^{-}\right]}_{i}^{R}\;if\;abs({\left[{Cl}^{-}\right]}_{i}^{S,min})\leq abs({\left[{Cl}^{-}\right]}_{i}^{S,max})$$

To quantify the influence of AMPA co-stimulation on the GABAergic [Cl^-^]_i_ transients, the amount of additional [Cl^-^]_i_ changes induced by AMPA co-stimulation (Δ_G_[Cl^-^]_i_) was calculated from the Δ[Cl^-^]_i_ values with/without AMPA co-stimulation as follows:

$$\Delta_{G}{\left[{Cl}^{-}\right]}_{i}=\Delta {\left[{Cl}^{-}\right]}_{i}^{AMPA/GABA}-\Delta {\left[{Cl}^{-}\right]}_{i}^{GABA}$$

## Supporting information

S1 Fig Effect of the membrane time constant on the trajectories of [Cl^-^]_i_ transients in a phase-plane plot. The black lines represent the [Cl^-^]_i_ changes induced by stimulation of GABA synapses only. Typical voltage deflections are displayed in the insets. Note that decreasing the membrane time constant (panel a) resulted in a comparable convergence of the trajectories towards 0 ms latency (purple line) and GABA only conditions (black lines) as under control conditions (panel b), despite the sharper trajectories. In contrast, after prolonging the membrane time constant (panel c) the trajectories did not converge to the 0 ms latency condition at the intersection with ∂[Cl^-^]_i_ /∂t = 0 mM/s (dashed line).
(TIF)

S2 Fig Effect of AMPA receptor activation on [Cl^-^]_i_ in a ball-and-stick model with a tonic GABAergic background conductance of 8.75 nS/cm^2^. Conductance and time constant of the AMPA receptor-mediated inputs were systematically varied as indicated in the diagram. Note the minimal [Cl^-^]_i_ changes in the μM range under these conditions. At physiological values for AMPA inputs (g_AMPA_ = 0.305 nS, τ_AMPA_ = 11 ms) a [Cl^-^]_i_ change of 0.1 μM was induced.
(TIF)

S3 Fig Effect of AMPA receptor co-stimulation on GABA_A_ receptor-induced [Cl^-^]_i_ transients in a morphologically realistic neuronal model with a tonic GABAergic background conductance of 8.75 nS/cm^2^. g_AMPA_ and [Cl^-^]_i_^0^ was varied as indicated in the graph, all other values were kept constant at physiological values (g_GABA_ = 0.789 nS, τ_GABA_ = 37 ms, τ_AMPA_ = 11 ms). Addition of the tonic GABA conductance had no obvious effect on the activity-dependent [Cl^-^]_i_ transients.
(TIF)

S4 Fig Effect of NMDA receptor co-activation on GABA_A_ receptor-induced [Cl^-^]_i_ transients in a morphologically realistic neuronal model. (a) Typical voltage deflections during a GDP at an [Cl^-^]_i_^0^ of 5 mM (left panel) and 25 mM (right panel) using different strength of NMDA co-stimulation as indicated by the color code. Note the substantial shift towards depolarized potentials at high g_NMDA_. (b) Time course of average dendritic [Cl^-^]_i_ at different [Cl^-^]_i_^0^ and g_NMDA_. Note that at both [Cl^-^]_i_^0^ the maximal [Cl^-^]_i_ change was augmented by addition of 107 NMDA synapses. (c) Statistical analysis of the voltage changes induced by simulated GDPs with different [Cl^-^]_i_^0^ and g_NMDA_. (d) Statistical analysis of [Cl^-^]_i_ changes induced by simulated GDPs with the given [Cl^-^]_i_^0^ and g_NMDA_ (closed bars) as compared to the [Cl^-^]_i_ changes with AMPA receptor co-stimulation (open bars). Bars represent mean ± SD of 9 repetitions.
(TIF)

S5 Fig Effect of AMPA receptor co-stimulation on GABA_A_ receptor-induced [Cl^-^]_i_ transients in a morphologically realistic neuronal model, in which the 107 AMPA synapses were positioned either in the distal or proximal quarter of each dendrite and the 534 GABA synapses in the remaining 75% of dendritic length to implement partial spatial decorrelation. Parameters are set to physiological values (g_GABA_ = 0.789 nS, τ_GABA_ = 37 ms, g_AMPA_ = 0.305 nS, τ_AMPA_ = 11 ms). (a) illustrates that the GABA_A_ receptor-induced [Cl^-^]_i_ transients are slightly altered with the reposition of the GABA synaptic sites. (b) The increase of [Cl^-^]_i_ transients by AMPA co-stimulation was virtually unaffected by this mild spatial decorrelation.
(TIF)
