# Supplementary figures and images for: Coincident glutamatergic depolarizations enhance GABAA receptor-dependent Cl- influx in mature and suppress Cl- efflux in immature neurons
Source: PLoS Comput Biol. 2021 Jan 19;17(1):e1008573. doi: 10.1371/journal.pcbi.1008573 (PMC7845986; doi:10.1371/journal.pcbi.1008573)

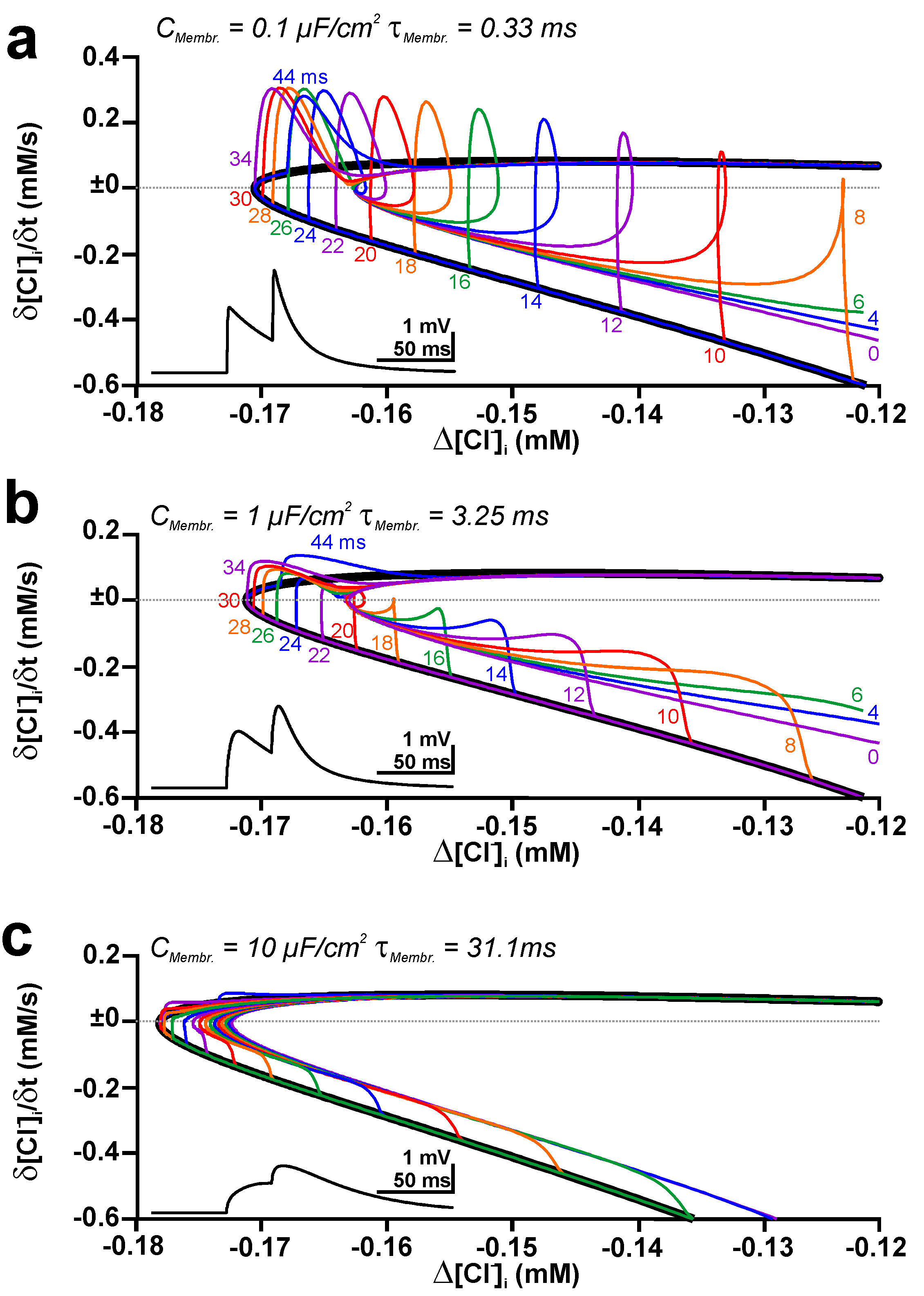

Supplement: S1 Fig — The black lines represent the [Cl-]i changes induced by stimulation of GABA synapses only. Typical voltage deflections are displayed in the insets. Note that decreasing the membrane time constant (panel a) resulted in a comparable convergence of the trajectories towards 0 ms latency (purple line) and GABA only conditions (black lines) as under control conditions (panel b), despite the sharper trajectories. In contrast, after prolonging the membrane time constant (panel c) the trajectories did not converge to the 0 ms latency condition at the intersection with ∂[Cl-]i /∂t = 0 mM/s (dashed line). (TIF) [file pcbi.1008573.s001.tif]

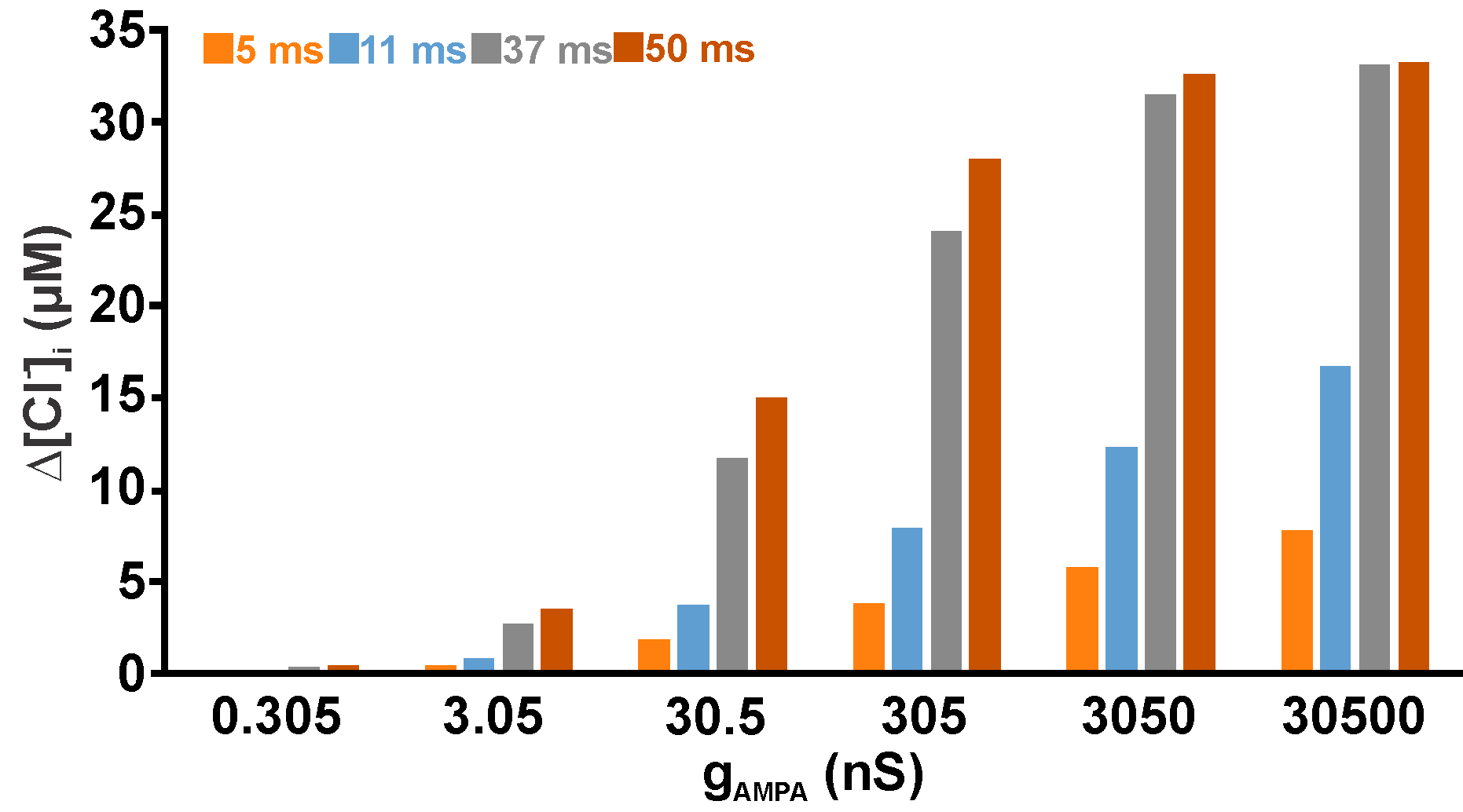

Supplement: S2 Fig — Conductance and time constant of the AMPA receptor-mediated inputs were systematically varied as indicated in the diagram. Note the minimal [Cl-]i changes in the μM range under these conditions. At physiological values for AMPA inputs (gAMPA = 0.305 nS, τAMPA = 11 ms) a [Cl-]i change of 0.1 μM was induced. (TIF) [file pcbi.1008573.s002.tif]

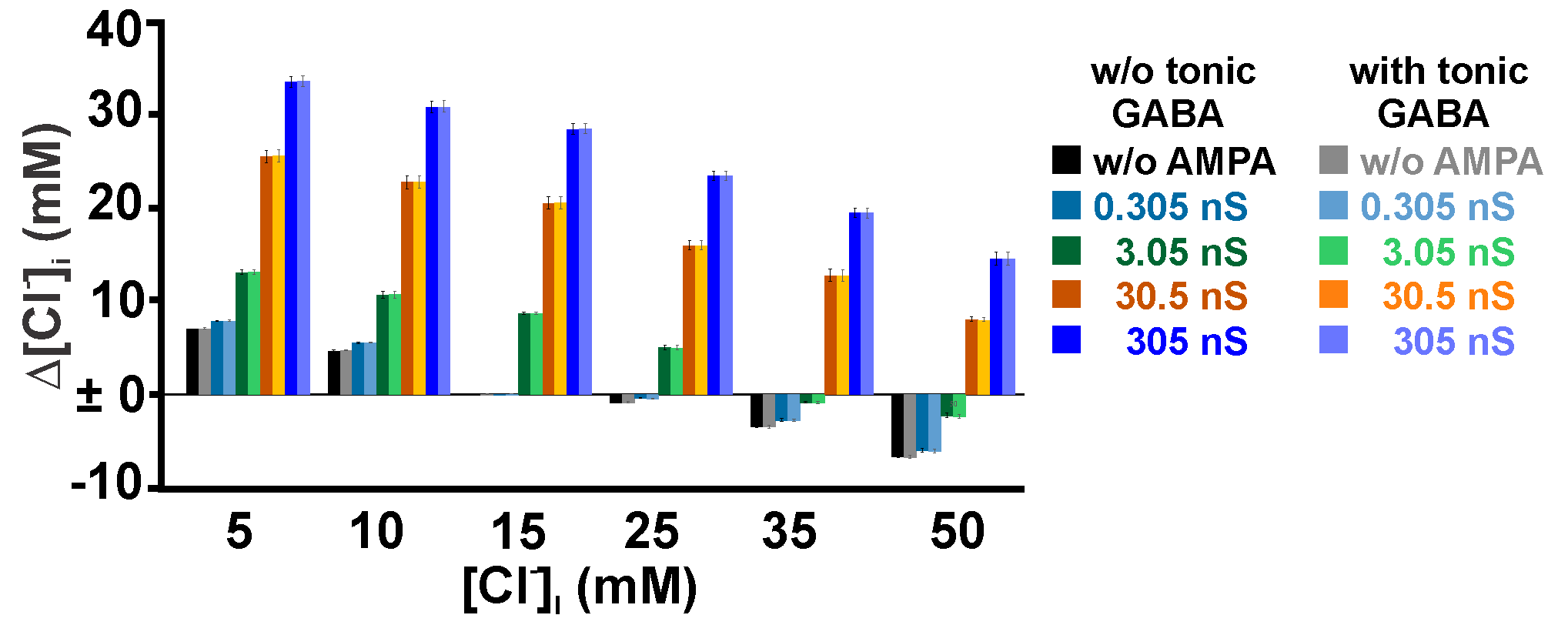

Supplement: S3 Fig — gAMPA and [Cl-]i0 was varied as indicated in the graph, all other values were kept constant at physiological values (gGABA = 0.789 nS, τGABA = 37 ms, τAMPA = 11 ms). Addition of the tonic GABA conductance had no obvious effect on the activity-dependent [Cl-]i transients. (TIF) [file pcbi.1008573.s003.tif]

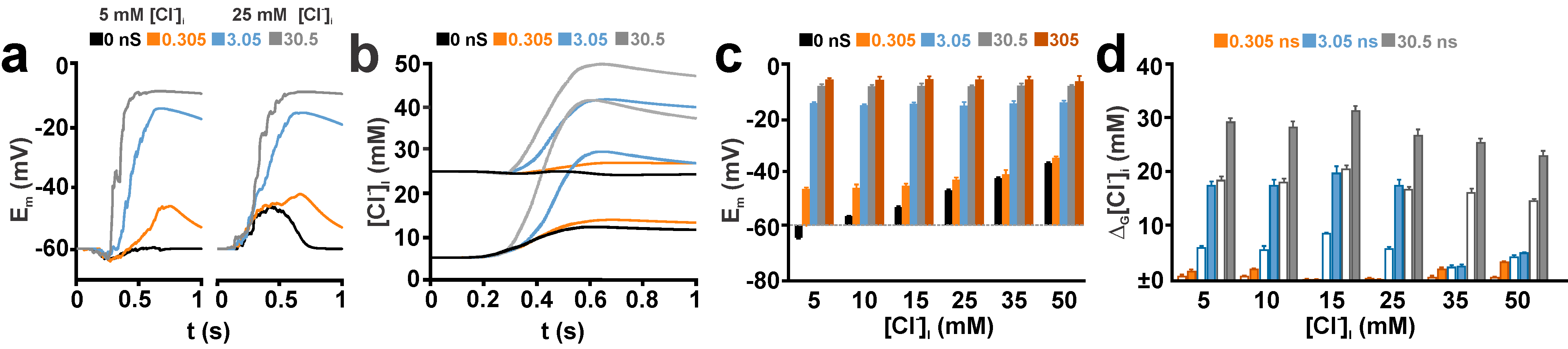

Supplement: S4 Fig — (a) Typical voltage deflections during a GDP at an [Cl-]i0 of 5 mM (left panel) and 25 mM (right panel) using different strength of NMDA co-stimulation as indicated by the color code. Note the substantial shift towards depolarized potentials at high gNMDA. (b) Time course of average dendritic [Cl-]i at different [Cl-]i0 and gNMDA. Note that at both [Cl-]i0 the maximal [Cl-]i change was augmented by addition of 107 NMDA synapses. (c) Statistical analysis of the voltage changes induced by simulated GDPs with different [Cl-]i0 and gNMDA. (d) Statistical analysis of [Cl-]i changes induced by simulated GDPs with the given [Cl-]i0 and gNMDA (closed bars) as compared to the [Cl-]i changes with AMPA receptor co-stimulation (open bars). Bars represent mean ± SD of 9 repetitions. (TIF) [file pcbi.1008573.s004.tif]

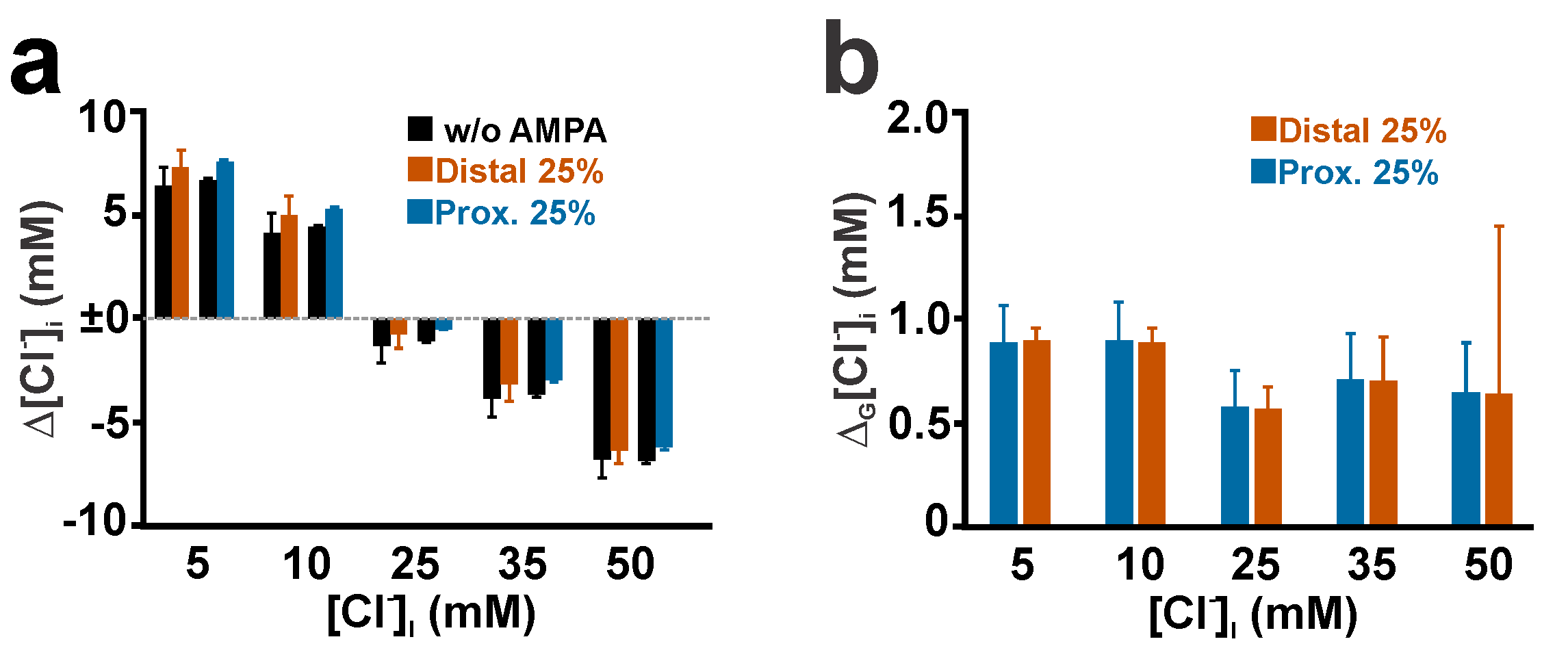

Supplement: S5 Fig — Parameters are set to physiological values (gGABA = 0.789 nS, τGABA = 37 ms, gAMPA = 0.305 nS, τAMPA = 11 ms). (a) illustrates that the GABAA receptor-induced [Cl-]i transients are slightly altered with the reposition of the GABA synaptic sites. (b) The increase of [Cl-]i transients by AMPA co-stimulation was virtually unaffected by this mild spatial decorrelation. (TIF) [file pcbi.1008573.s005.tif]
