# Supplementary material for: Coincident glutamatergic depolarizations enhance GABAA receptor-dependent Cl- influx in mature and suppress Cl- efflux in immature neurons
Source: PLoS Comput Biol. 2021 Jan 19;17(1):e1008573. doi: 10.1371/journal.pcbi.1008573 (PMC7845986; doi:10.1371/journal.pcbi.1008573)
Supplement: Attachment — Submitted filename: Lombardi et al_Response_to_Reviewers_Final.pdf [file pcbi.1008573.s007.pdf]

Responses to Reviewers:

Reviewer #2: *The authors perform a systematic exploration of how GABA and Glutamate activations at different time points and locations affect the intracellular Cl- concentration in a hippocampal neuron. This is relevant because the GABA-current depends on the transmembrane concentration gradient of Cl-, and changes in intracellular Cl- leads to a so-called "ionic (synaptic) plasticity" of GABA synapses.*

The results section consists of a large number of simulations where different parameters (locations, timing, synaptic conductances, synaptic time constants, the membrane time constant, initial Cl- concentration) are varied, mostly one (or two) at the time. It is not easy to boil all that down to any general take-home message, since the effects will always depend on this or that, but I suppose that a general take-home message might not be expected for an intricate system like this.

The main finding seems solid, though, and is reflected in the manuscript title. When the initial Cl- concentration is low/intermediate (mature neurons), Glutamatergic depolarizations tend to enhance Cl- influx during GABAergic activation. This causes an "ionc plasticity" that decreases the inhibitory action of GABA. Differently, when the initial Cl- concentration is high (immature neurons), GABA activation has a depolarizing effect. Glutamatergic depolarizations then tend to suppress Cl- efflux during GABA-activation, thus preserving the ion reservoir (battery), and stabilizing the deploarizing role of GABA.

The modeling work seems solid, and the paper is well written. I think it is suited for publication in PLoS CB, but I have a some (relatively minor) suggestions to how it might be improved:

We thank the reviewer for the supportive comments on our manuscript.

Point 1: I get the feeling that the paper could be shortened and written in a way that puts more focus around the key findings. Some of the systematic parameter explorations are nice to include, but do not always lead to all that interesting results. Parts of it might be hidden away as supplementary material and simply summarized in the body text. I do not regard this as a criterion for publication though – just a suggestion that the authors could consider.

In order to address this suggestion, we removed some panels from the previous version of the manuscript (illustrating the influence of membrane time constant on the phase plane trajectories [former Fig. 6b]) and included part of the new experiments requested by the reviewers as supplementary figures.

Point 2: As Fig. 4 (not surprisingly) shows, the Glutamate-effect on Cl- during AMPA activation is solely due to the membrane depolarization that it evokes. The question is then: Is the EPSP from AMPA-activation generally the main source of membrane depolarization in the dendrites of

CA3-neurons? What about back-propagating action potentials, dendritic Ca2+ spikes, or (local) secondary effect of AMPA activation mediated by locally present ion channels? Would these phenomena play an equally important role for ionic plasticity?

I think this issue should be addressed, at least as a passage in the discussion. Perhaps it is beyond the scope of the current paper to actually explore such effects, but I guess it would be possible to do so, using some existing CA3-neuron model with active dendrites, such as the one by Traub et al. 1991 [Journal of Neurophysiology. 1991; 66(2):635–650], Pinsky & Rinzel 1994 [Journal of computational neuroscience. 1994; 1(1-2):39–60], Migliore et al. 1995 [Journal of neurophysiology, 73(3), 1157-1168.], Hemond et al. 2008 [Hippocampus, 18(4), 411-424], or Sætra et al. 2020 [PLoS Comput Biol 16(4): e1007661], the latter of which also includes ion concentration dynamics.

The reviewer is absolutely right, that in addition to glutamate receptors also other depolarizing events should contribute to a modulation of the GABA-mediated ionic plasticity. Therefore, we decided to address these suggestions also in the revised manuscript. However, due to a lack of time and space in the manuscript, we were not able to address these points with the same analytical rigor as we did for the AMPA receptors.

We investigated the effect of NMDA receptors on GABA-induced $[Cl^-]_i$ transients in the ball-and-stick model and the reconstructed neurons. For this purpose, we implemented an established model for NMDA receptors (Senselab Model DB ID: 145836, Moradi et al., 2013, J Comput Neurosci, see lines 753-755). These simulations revealed for the ball-and-stick model that co-activation of NMDA receptors influenced the GABA-induced $[Cl^-]_i$ transients with a comparable maximal effect as AMPA, but with a steeper dependency from g_{NMDA} (lines 239-247, Fig. 3e). For the reconstructed neuron, the GABA-induced $[Cl^-]_i$ transients were in general stronger augmented by co-activation of NMDA receptors than by AMPA receptors (lines 502-509, Supl. Fig. 4). These results are discussed in lines 647-657.

In addition, we also addressed the question, whether Ca^{2+} channels, somatic, and/or dendritic action potentials can augment GABAergic $[Cl^-]_i$ transients. For this purpose, we implemented an established model incorporating voltage gated Ca^{2+} channels and Hodgkin-Huxley processes (Model DB ID: 3263, Migliore et al., 1995, J. Neurophys, see lines 795-801) in the ball-and-stick model. These simulations revealed that the addition of Ca^{2+} conductances had only a negligible effect on the activity-dependent $[Cl^-]_i$ changes. Moreover, HH mechanisms in the soma enhanced the $[Cl^-]_i$ changes only at high GABAergic conductances (and thus large, suprathreshold depolarizations), but implementing HH mechanisms in the dendrite generated substantially larger $[Cl^-]_i$ changes upon GABA activation (line 248-258, Fig. 3f-h). These results are discussed in line 657-661.

Point 3: [Ca-]i is used in two different meanings - it denotes both the variable intracellular concentration (e.g., on the y-axis in Fig. 1C) and the (constant) initial condition (e.g. in the legend of Fig. 1b). You should make a distinction, and perhaps add an index "0" when the latter is meant.

In order to follow the reviewer's comment, we now used $[Cl^-]_i^0$ to denote the initial Cl^--concentration throughout the manuscript.

Point 4: Line 134 refers to the "passive [Ca-]i ". It sounds odd to call a concentration passive. Perhaps "baseline" is better?

To follow the reviewer's suggestion, we now used the term "$[Cl^-]_i$ given by a passive distribution at -60 mV" instead of "passive $[Cl^-]_i$" (line 134). Please note that the passive distribution is not necessarily the "baseline" $[Cl^-]_i$, as this value is determined by Cl-transport, basal Cl^--conductances as well as ongoing tonic and phasic GABAergic activity.

Point 5: Line 157 reads: "These simulations revealed that without co-stimulation GABAA receptor induced Cl- fluxes depended only on the initial [Cl-]i (Fig. 2a)." I don't think anything is revealed here. The initial [Cl-]i is the only thing being varied, so it could not have depended on anything else?

To follow the reviewer's concern, we rephrased this sentence to: "As expected, these simulations showed that $GABA_A$ receptor induced Cl^- fluxes depended on the $[Cl^-]_i^0$ (Fig. 2a black trace)" (Line 159).

Point 6: Line 344: When you write "speeding up the kinetics of AMPA and GABA-receptor dependent voltage responses", you make me think that you change the AMPA and GABA time constants, but here I think you have changed the membrane time constant, which is a more general "speeding up of things". I think you should rephrase this to avoid confusion.

In order to follow the reviewer's concern, we rephrased this sentence to:" These simulations revealed that reducing the membrane time constant, which enhanced the onset kinetics of AMPA and GABA receptor- as well as the decay of AMPA receptor-mediated voltage responses, had only a minor effect on the observed $[Cl^-]_i$ changes" (line 381-384).

7. Line 364: You refer to your "full" model as "morphologically and biophysically realistic", although it is a passive model with no ion channels, so it is unclear what kind of biophysical realism you make claim to. How is it more biophysically realistic than e.g., the ball-and-stick model – they differ solely in terms of morphology?

In order to follow the reviewer's suggestions, we now avoid the term "morphologically and biophysically realistic" and replaced it with the more adequate term "morphologically realistic" (Lines 411, 414, 453, 511, 597)

8. Line 636: "the dendrite was electrically and diffusionally detached from the soma". This means that the model, in practice, did not have any soma, so you did not use a ball-and-stick model, but a stick model. If you only modelled the dendrite, it should be made clear also in the Results-section. Also, I am a bit surprised by this choice. Why cut off the soma? Wouldn´t you expect more realistic results with it being present?

In order to follow the reviewer's comment, we re-calculated all simulations in the reduced morphology using a ball-and-stick model and omitted the "isolated dendrite configuration". All values and figures are changed to this new values. Note that we did not redline these changes. In addition, we removed the objected sentence from the materials and methods section.

9. In dilute solutions, the diffusion constants for Cl- and HCO3 are 2.03 µm2/ms and 1.18 µm2/ms, respectively (Table 2.1 in Grodzinsky F. Fields, Forces, and Flows in Biological Systems. Garland Science, Taylor & Francis Group, London & New York.; 2011). They could be smaller than that in the cytoplasm (due to obstacles), but I do not think it is possible that they become any larger. Hence, using a value of 2 µm2/ms for HCO3 (Line 675) is probably incorrect. This is not likely to have any severe impact on the simulation results, I imagine, but if it is not too much work, you might want to fix it.

In order to follow the reviewer's comment, we now repeated all simulations using a diffusion coefficient of 1.18 $\mu m^2/ms$ for HCO_3^- ions (Kigoshi K. and Hashitani T. (1963) Bull. Chem. Soc. Jpn. 36(10), pp. 1372– 1372) (see line 810 in the methods). All values in the text and all figures are updated. Please note that we did not redline these altered values, to ease the recognition of all other changes in the manuscript.

<u>Reviewer #3:</u> *This paper uses computational methods to explore how AMPA mediated glutamatergic input affects GABAAR mediated Cl- dynamics in neurons. The paper is well written, clear and logically developed. Although the results perhaps aren't "earth shattering" or hugely unexpected over what one would predict conceptually, I found the work to be broadly satisfying and certainly publication worthy in this journal;. I found the modelling of GDPs in morphologically realistic immature CA3 neurons particularly good. I also enjoyed the attention payed to how glutamatergic conductances affect neurons with both low and high initial Cl- concentrations. Compared to some other computational models of Cl- dynamics the authors used a rather simple mechanism to describe Cl- extrusion/ intrusion by KCC2/NKCC1, this is not necessarily a problem and I can see how this would have aided the ease of computation in the more morphologically realistic neurons, but I think the authors do need to be a bit more upfront about some of the limitations of the model.*

We thank the reviewer for the detailed evaluation of our manuscript and his/her helpful comments. In line with the suggestion of the reviewer regarding the limitations of the simple Cl- extrusion/ intrusion mechanism by KCC2/NKCC1, we added a brief description of the limitations of the used model (line 603-614).

Major concern 1: In the author's model, they use a passive conductance (gpas) that does not include Cl- flux. This is despite the fact that tonic Cl- conductances (including tonic GABAAR) exist in neurons. As a result the authors by definition omit a relevant possible route whereby glutamatergic conductances can change [Cl-]I via affecting the driving force for Cl- flux across GABAARs. For example, in section 2.2 which investigates the influence of τAMPA on GABAA receptor induced [Cl-]i transients AMPA conductances with taus longer than the GABAA taus have no effect on the change in [Cl-]I this makes sense in their model as once the GABAAR conductance is over the AMPA driven change on the GABAAR driving force can no longer affect Cl- flux as there is nor GABAAR conductance. In most neurons though tonic GABAAR exists and hence the longer AMPA conductances should progressively change [Cl-]i (not as much as during the phasic GABAAR conductance, but still a bit!). The authors need to address this issue and either model tonic GABAAR, or justify why they did not. Ie Either they need to explicitly ref the fact that tonic GABAAR is small / minimal in immature CA3 neurons hence why they excluded tonic GABAAR conductances and acknowledge that tonic GABAAR in other cell types could affect their findings / make some predictions – ie what would happen in cells with high tonic GABA? Or they need to do some simulations where tonic Cl- flux is also modelled to explore how this variable affects glut-mediated Cl- changes via GABAARs. Ie what is the difference between cells with low tonic GABAA vs high tonic GABAA - for example some version of the simulations in section 2.2, but where tonic Cl- flux is present (there are various ways this could be modelled.) New simulations are not essential but I think attention to this would certainly strengthen the paper.

We thank the reviewer for pointing out this issue. The original intention of this study was to evaluate the direct interactions between glutamatergic and GABAergic synaptic inputs to elucidate whether they contribute to ionic plasticity. Thus we tried to make the conditions as strict as possible. However, as the reviewer points out that tonic GABAergic conductances can mediate $[Cl^-]_i$ fluxes that contribute to activity dependent ionic plasticity, we decided to include them into this manuscript. We therefore performed additional simulations using both, ball-and-stick model and the reconstructed neuron, in which we included a tonic GABAergic conductance (see line 743). However, simulations of these tonic GABAergic conductances revealed that activation of AMPA receptors mediates only minimal additional AMPA-mediated $[Cl^-]_i$ changes (line 398-408, Supl. Fig. 2) and only marginally affects the $[Cl^-]_i$ changes induced by AMPA-GABA co-activation (line 408-409, Fig. 6e). Accordingly, also in the reconstructed neuron addition of a tonic GABAergic conductance had only a negligible effect on the activity-dependent $[Cl^-]_i$ changes (lines 445-450, Supl. Fig. 3). We discussed these results in line 660-661. Since these experiments indicate that tonic GABAergic currents had in our models only a negligible effect and to reduce computational complexity of our models, we did not implement a tonic GABAergic current for all further simulations.

Major concern 2: The second limitation of the model which the authors do not state explicitly is the fact that NMDA receptors are not modelled, just AMPA receptors. The authors need to either state why this was the case, or acknowledge this as a limitation? For example how does NMDA change the ball-and-stick model's results. I realise that τAMPA was changed up to 100 ms. But NMDA does have different kinetics, including reliance on Vm.

In order to follow the reviewer's suggestion (see also point 2 of reviewer #2), we investigated the effect of NMDA receptors on GABA-induced $[Cl^-]_i$ transients in the ball-and-stick model and the reconstructed neurons. For this purpose, we implemented an established model for NMDA receptors (Senselab Model DB ID: 145836, Moradi et al., 2013, J Comput Neurosci, see lines 792-800). These simulations revealed for the ball-and-stick model that co-activation of NMDA receptors influenced the GABA-induced $[Cl^-]_i$ transients with a comparable maximal effect but a steeper dependency from g_{NMDA} (lines 239-247, Fig. 3e). For the reconstructed neuron the GABA-induced $[Cl^-]_i$ transients were in general stronger augmented by co-activation of NMDA receptors than by AMPA receptors (lines 502-509, Supl. Fig. 4). These results are discussed in lines 646-660. However, due to limitations in time and space (of the text) we did not investigate this topic in more detail.

Major concern 3: In addition active voltage-gated conductances also weren't modelled. In the discussion a sentence should be added about how these are likely to add further complexity / another mechanism which could enhance the ability of glutamatergic input to depolarize the neuron and exacerbate Cl- influx via GABAARs.

In order to follow the reviewer's suggestion (see also point 2 of reviewer #2) we also added an investigation how active voltage-gated conductances contribute to the $[Cl^-]_i$ changes. In particular, we addressed the question, whether Ca^{2+} channels, somatic, and/or dendritic action potentials can augment GABAergic $[Cl^-]_i$ transients. For this purpose, we implemented an established model incorporation voltage gated Ca^{2+} channels and Hodgkin-Huxley processes (Model DB ID: 3263, Migliore et al., 1995, J. Neurophys, see lines 795-801) in the ball-and-stick model. These simulation revealed that the addition of Ca^{2+} conductances had only a negligible effect on the activity-dependent $[Cl^-]_i$ changes, that HH mechanisms in the soma enhanced the $[Cl^-]_i$ changes only at high GABAergic conductances (and thus large, suprathreshold depolarizations), but that implementing HH mechanisms in the dendrite generated substantial larger $[Cl^-]_i$ changes upon GABA activation (line 248-258, Fig. 3f-h). These results are discussed in line 657-661.

Minor point 1: I think an important thing to think about is the colours used in the images, which may be difficult to interpret, especially for those with colour blindness. Here are some colour palettes (from seaborn) and their performance with different vision deficiencies:

We thank this reviewer for this helpful comment. We did not consider this issue before. In order to improve the readability of our manuscript we changed all figures according to the Seaburn palette “Colorblind 10”.

Minor point 2: I thought the final sentence of the discussion was too strong and not justified by the papers findings. “These glutamatergic modulations of GABAergic ionic plasticity can contribute to short-term memory and may profoundly influence information processing in the developing and mature nervous system.” Change to: “These glutamatergic modulations of GABAergic ionic plasticity could possibly contribute to short-term memory and are likely to influence information processing in the developing and mature nervous system.”

We changed this sentence in accordance with the reviewer’s suggestion to: “These glutamatergic modulations of GABAergic ionic plasticity could possibly contribute to short-term memory and are likely to influence information processing in the developing and mature nervous system.” (line 730-732).

And finally we corrected the typos found by this reviewer:

Typos:
Line 360: gAPMA -should be gAMPA
Line 381 (i.e. the experimentally determined number and conductance of GABAergic inputs) - should be AMPA inputs
Line 603: inhibition at will – remove “at”

<u>Reviewer #4</u>*: I congratulate the authors for their manuscript.*
The authors invetigate how coincident excitatory input shapes inhibition and more precisely how Cl- flux associated with gaba currents and subsequent change in chloride concentration is modulated by concurrent excitation. This phenomenon is explained by the fact that excitatory currents depolarize the membrane, which increases the Cl driving force and shapes Cl flux.
The most impressive part of the manuscript is the systematic manner in which the authors investigate the effect of synaptic events time constant, location and synchronisation on Cl- fluxes. They performed a large number of simulations and made extensive anaysis. In particular to my knowledge, the impact of Ampa time constant on Cl- accumulation has not been investigated before.
The topic is important as the results shed light on the determinants of inhibitions and may help better understand how to restore impaired inhibition in pathologies.

That said, the redaction and presentation could be improved at some places. I give here a list specific comments:

We thank the reviewer for the detailed evaluation of our manuscript and his/her helpful comments.

Point 1: In the abstract, the author mention that they model immature CA3 neurons. As chloride homeostasis is strongly influenced by maturity, it would be nice if the authors motivated their choice to model an immature neuron.

The reviewer raises an important point here. We picked this reconstruction of an immature hippocampal neuron because in the second part of the manuscript we modelled ionic plasticity by using coincident GABAergic and glutamatergic activity recorded in an immature hippocampal CA3 neuron (taken from Lombardi et al., 2018). Thus we considered that a reconstruction of such a neuron (actually the reconstructed neuron is one of the neurons we recorded this immature coincident AMPA/GABA activity burst from) is best suited to model ionic plasticity under our conditions. In order to follow the reviewer's concern, we now included this motivation in the manuscript (line 745-747 in the methods and shortly in line 419 in the results section). Since, we were not able to include this reasoning already in the abstract and to avoid confusion, we also changed the phrase "reconstructed immature CA3 neuron" to "reconstructed CA3 neuron" in the abstract (line 32).

Point 2: At page 2, lines 43-44, I don't understand the mean of 'destabilization' in the following: 'enhancing the destabilization of GABAergic inhibition in the mature nervous systems'

In order to follow the reviewer's suggestion, we replaced the term "destabilization" with "attenuation". The objected sentence now reads: "… our results demonstrate that glutamatergic co-stimulation had a substantial impact on ionic plasticity of GABAergic responses, enhancing the attenuation of GABAergic inhibition in the mature nervous systems…" (lines 42-44).

Point 3: At the beginning of introduction, line 63. The expression 'Information processing between neurons' is strange. A single neuron processes information but information is transfered between neurons through synapses. There is a disctinction between processing and transfering information.

In order to follow the reviewer's suggestion, we changed the objected phrase to "Information transfer within networks of single neurons" (line 63).

Point 4: Introduction line 70: 'with a high Cl- permeability and a partial HCO3 permeability'. It is strange to contrast 'high' with 'partial' instead of 'high' with 'low' or 'full' with 'partial'

In order to follow the reviewer's comment, we changed this sentence to: "… are ligand-gated anion-channels with a high Cl- permeability and a lower HCO_3^- permeability…" (Line 71)

Point 5: Line 71, what does 'gaba actions' mean? This is a strange wording. Can actions be replaced by a more specific term

As we refer here to a variety of consequences of $GABA_A$ receptor activation, like hyper-/depolarization or inhibition/excitation, we would like to maintain a global, unspecified term here. Please note that we resolved the exact meaning of GABAergic effects in the following sentences of the manuscript. However, we changed the term "GABA actions" to "GABAergic effects" as we now feel that this term is probably less problematic than the previous term "GABA actions" (line 71).

Point 6: Line 75, 'the Na+-dependent K+-2Cl--Symporter NKCC1...' it was my belief that the stochiometry of NKCC1 is rather Na+-K+-2Cl-

To avoid further confusion, we replaced the term "Na^+-dependent K^+-$2Cl^-$-Symporter" with the better defined term "Na^+-K^+-$2Cl^-$-Symporter" (line75).

Point 7: Page 6, lines 123-124. 'leads' and 'allowed' should have the same verb tense.

We changed "leads" to "led" in line 123.

Point 8: Line 131, DFGABA should be formally defined.

In order to follow the reviewer's suggestion, we defined the electromotive force at the first instance it was introduced to the text (line 131)

Point 9: Figure 1 a. Here and elswhere, what is shown in the schematic next to GABA and AMPA? is it the time course of current, of conductance or something less? It would be nice to indicate it on the schematic. Scale bars could be added.

In this small inset in the schematic representation of the experiments the GABA- and AMPA-mediated currents are schematically depicted. We now explicitly mention this in the legends to Fig. 1A (line 146), Fig. 3A (line 215), and Fig. 4A (line 282). However, as these are schematic representations and as a scale will be hardly readable within this small panels, we did not add a scale bar to them.

Point 10: Figure 2 a and d. These panels are somewhat difficult to understand quickly because of the broken lines corresponding to biphasic responses. The double blue curves in a could look at first glance like a mistake. Maybe there is another way to display the results? Maybe making two different panels for Cl influx and efflux.

In order to follow the reviewer's comment, we now separated the $[Cl^-]_i$ response curve in two independent panels for Cl^--influx and -efflux in the figures Fig.2e+f and Fig3d+e+h.

Point 11: Comments 9 and 10 are also valid for figure 3.

See our comments to Points 9 and 10.

Point 12: Page 12, lines 235-237. Where is Em recorded? Is it always somatic Em? This could be specified.

We thank the reviewer for noticing this important neglect in our manuscript. For the ball-and-stick model we recorded Em as well as $[Cl^-]_i$ at the site of the GABAergic synapse. This was now explicitly described in the materials and methods (line 823-826) and was mentioned in the results part (lines 147 and 151). For the reconstructed neurons we averaged $[Cl^-]_i$ within the dendritic compartment (Except for Fig. 7d), as already described in the previous version of the manuscript (line 425-427). However, we now added the information that Em was determined in the soma (line 427 and 824-825).

Point 13: Line 240-241, the definition of Delta_G[Cl-]i is difficult to read as an inline equation. Maybe it could be defined in text with a full sentence.

In order to follow the reviewer's suggestion, we rephrased these sentences as follows: "To quantify this effect, we calculated the amount of additional $[Cl^-]_i$ changes induced by AMPA co-stimulation ($\Delta_G[Cl^-]_i$) by subtracting the $[Cl^-]_i$ changes at GABA stimulation from the $[Cl^-]_i$ changes at AMPA/GABA co-stimulation." (line 270-272).

Point 14: Page 13, lines 256-257. Is the decrease really exponential? If so, could you explain why? Did you try to fit an exponential and find the constant of the exponential and goodness of fit?

Yes, we fitted this decay with a monoexponential function, so it can be sufficiently described with a mono-exponentially function (the small R^2-Value of 0.023 (using n= 100 data points) indicate that any additional exponential component will only have a minimal impact on the goodness of the fit). The constant of this exponential fit has already been given in the previous version of the manuscript as length constant (now line 274-275). We now also included a value for the goodness of the fit (R^2 = 0.023). This exponential decay simply reflects the length constant of a passive cable. To follow the reviewer's question, we mention this hypothesis in the manuscript in line 276-277 as follows" … and reflects the exponential decay of voltage in a linear cable with homogenic membrane resistance".

point 15: Line 263. Why did you choose the range -49ms-100ms?

We randomly set this value range. When the AMPA pulse started 49 ms before the GABA pulse a residual AMPA current component of 1.1% remained (for a tau-AMPA of 11 ms). Thus we assume that a latency of 49 ms is sufficient for a clear separation of AMPA and GABA inputs. After 100 ms a residual GABA current of 6.7 % remained, indicating that only small $[Cl^-]_i$ changes would be expected under this conditions. Please consider, that each point in these curves represents the analysis of a separate simulation, therefore we did not explore the temporal relations beyond these, in our opinion, sufficiently large intervals.

Point 16: Figure 5 a and b lower parts. I dont like when several curves are superimposed as it provides little information. Could you provide the difference between the curves so we can see how much they are really alike?

In order to follow the reviewers comment, we now plotted the differential $[Cl^-]_i$ changes in the Figs 5a and b (bottom panel). However, we decided to keep the original $[Cl^-]_i$ traces, as they emphasize that relative small size of the effect of AMPA on the $[Cl^-]_i$ changes.

Point 17: Page 16 line 320. What exactly do you mean by complex influence? Could you be more specific?

In order to follow the reviewer's concern, we replaced the term "complex influence" with a more detailed description as follows: From these results it can be concluded, first, that the effect of

AMPA co-stimulation on $\Delta_G[Cl^-]_i$ depends critically on the timing between both inputs, second, that $\Delta_G[Cl^-]_i$ is maximal when τ_{GABA} is slightly larger than τ_{AMPA}, and, third, that a "plateau"-like interval of stable $\Delta_G[Cl^-]_i$ occurred when τ_{GABA} is at least 3 times larger than τ_{AMPA}." (line 356-360).

Point 18: Page 17, line 340. What does voltage 'deflections' mean? Could you use a more specific word.

In order to follow the reviewer's suggestion, we now changed this sentence to "…, we investigated how the time course of E_m changes contributes to this complex dependency" (line 378).

Point 19: I find figure 7 impressive!!

Thank you!

Point 20: In the legend of figure 7, a line break occurs in the middle of [Cl-]i, this should be corrected.

We corrected this issue throughout the manuscript (not redlined).

Point 21: When plus-minus values are given, are they standard deviations? It is not clear what is the random part of the simulation. Is the position of the synapses random? Is the timing of synaptic events random? this could be better explained

In order to follow the reviewer's concern, we (i) provided the information that the quantitative values for the reconstructed cell are gives as Mean ± SD (line 775-778) and (ii) describe the procedures used for random distribution of synaptic inputs in detail (lines 779-791). To make this information more accessible, we also shortly describe the procedures of random GABAergic stimulation already in the results section (lines 429-431).

Point 22: Page 22, lines 455-456: 'Therefore, we next located the GABA and AMPA synapses in different parts of the dendritic compartment.' What does it exactly means? On the same dendritic branch but at different distances from the soma or on different dendritic branches?

To follow the reviewer's concern, we now explicitly describe the localization of the synapses in these two configurations as follows: "Therefore, we next relocated the GABA and AMPA synapses in distinct parts of the dendritic compartment, with either AMPA synapses located only in the most distal 12 dendritic branches and the GABA synapses in the most distal 34 dendritic branches, or vice versa (Fig. 9b)" (line 523-525).

Point 23: Figure 9a, bar graph. Is there any difference between the red and blue bars or are they exactly the same? If they are not exactly the same, the authors should find a way to illustrate the difference. From what I understand, each black bar is repeated twice with pairs of black bars having the exact same value. This should be avoided.

In order to follow the reviewer's suggestion, we replaced the $\Delta[Cl^-]_i$ bar diagram with a bar diagram illustrating $\Delta_G[Cl^-]_i$ in Fig. 9b, thereby omitting the black bars (representing GABA alone stimulation).

Point 24: Fig 9e. It is extremely hard to decipher this panel.

In order to follow the reviewer's concern, we now provide a short description of the cross-correlograms in the legend to this panel as follows: "The relative occurrence of AMPA stimuli in relation to each GABA stimulus was plotted, demonstrating the lack of AMPA receptor mediated synaptic input in the refractory intervals" (line 551-553).

Point 25: Page 23. Line 485-487. The authors should explain in more details what is random in their simulation and eventually what random distribution is used and why.

In order to follow the reviewer's concern, we now added this important information to the materials and methods section (lines 779-791) and a short description to the results section (line 561).

Point 26: Page 26 line 523. I don't understand what 'stringent' means in this context. Maybe use a more technical and specific word.

We replaced the word "stringent" by the word "substantial" (line 595).

Point 27: Line 533, when the authors mention inhibition, do they mean hyperpolarization or decrease in spike rate? This could be specified.

Within our scope, inhibition mainly refers to an increase in the rheobase (i.e. in the amount of excitatory currents required to trigger an action potential). For networks, this can be best interpreted as a decrease in spike probability. In order to follow the reviewer's suggestion, we now mentioned the definition of inhibition in our manuscript and how it is related to hyperpolarization in the discussion (line 619-621).

Point 28: Page 26 line 555. The authors mention coding. The notion of coding in this context is quite complex and subtle. The notion of coding could be exposed and explained a bit.

In the single neuron perspective, the term "temporal coding" is sometimes used to describe the optimal temporal correlation between excitatory and GABAergic inputs. Therefore, we explained this issue in the manuscript as follows: "but may also affect the temporal fidelity of inhibitory synaptic inputs, as with a weakening of GABAergic inhibition the temporal window for effective inhibition of excitatory inputs became smaller" (line 643-645). However, a detailed discussion of this issue is beyond the scope of this manuscript.

Point 29: Page 27, line 561. The meaning of 'moderate' is not clear for me.

To be more precise here, we avoid the term "moderate" and rephrased this sentence as follows: "our computational model demonstrates that glutamatergic co-stimulation at physiologically relevant levels (g_{AMPA} of 0.305 to 3.05 nS, corresponding to 1 to 10x of experimentally determined conductance for spontaneous AMPA receptor mediated inputs) caused biphasic $[Cl^-]_i$ changes at $[Cl^-]_i$ values between 15 and 35 mM (i.e. in the typical range determined in immature neurons [4,57,58]) (line 664-668).

Point 30: Methods line 646. Why not use GHK flux equations which is more accurate when ionic concentrations fluctuate.

Although we agree that a GHK model will provide more accurate results, for the relatively small $[Cl^-]_i$ changes this ohmic model provides a sufficiently precise estimate of driving forces (see also Jedlicka et al. Hippocampus 2011 for the similarity of GHK and ohmic results). Since we have to model both, dynamic $[Cl^-]_i$ and $[HCO3^-]_i$ it was required to calculate I_{Cl} and I_{HCO3} separately. And as we calculated both, E_{Cl} and E_{HCO3}, for each dt from the extra and intracellular concentrations using the Nernst equation, we also handle the fluctuations in$[Cl^-]_i$ and $[HCO3^-]_i$. Therefore, we decided to maintain our model for GABAergic ion fluxes and internal Cl^- and HCO_3^- handling.

Point 31: Page 31, line 663. What exactly follows a Gaussian distribution? Is it the time separating two consecutive onsets of synaptic events?

No, these are the exact time points at which synaptic input occurs. To follow the reviewer’s concern, we rewrote the respective sentence in the materials and methods as follows: “The time points of GABA and AMPA inputs were determined stochastically using a normal distribution…” (line 780-784). With this Gaussian distribution we are able to emulate the time course of synaptic inputs during a GDP (see Fig. 7c).

Point 32: Page 32. Line 690. The long superscript is hard to read. Maybe the equation could be reformated.

To follow the reviewer’s concern we omitted this formula and described the averaging procedure in the text (line 825-826).

Point 33: Page 33, Lines 697-698. 'abs' should not be in italic.

We changed this according to the reviewer’s suggestion (Lines 833, 834)

Point 34: Is the formal definition of DFGABA given somewhere in the manuscript?

In order to follow the reviewer’s suggestion, we defined the electromotive force at the first instance it was introduced to the text (line 131).

Point 35: In the abbreviations, the PHCO3 is the relative permeability. Relative to what? This should be written explicitly

As we did not use this abbreviation in the manuscript, we omitted it from the abbreviation list. The definition of the “relative bicarbonate permeability” p had already been given in the manuscript in line 759.
